# The urokinase‐type plasminogen activator system as drug target in retinitis pigmentosa: New pre‐clinical evidence in the rd10 mouse model

**DOI:** 10.1111/jcmm.14391

**Published:** 2019-06-28

**Authors:** Maurizio Cammalleri, Massimo Dal Monte, Filippo Locri, Valeria Pecci, Mario De Rosa, Vincenzo Pavone, Paola Bagnoli

**Affiliations:** ^1^ Department of Biology University of Pisa Pisa Italy; ^2^ Department of Experimental Medicine Second University of Napoli Napoli Italy; ^3^ Department of Chemical Sciences University of Napoli Federico II Napoli Italy

**Keywords:** apoptosis, autophagy, cone arrestin, ERG, Müller cell gliosis, pro‐inflammatory markers, retina degenerative disease, rod markers, UPARANT (Cenupatide), αvβ3 integrin/Rac1 pathway

## Abstract

Retinitis pigmentosa (RP) is characterized by progressive loss of vision due to photoreceptor degeneration leading to secondary inflammation. The urokinase‐type plasminogen activator (uPA) system contributes to retinal inflammation, but its role in RP is unknown. In the rd10 mouse model of RP, we addressed this question with the use of the peptide UPARANT designed to interact with the uPA system. UPARANT was systemically administered from post‐natal day (PD) 10 to PD30 when its efficacy in RP rescue was investigated using electroretinographic recordings, Western blot and immunocytochemistry. Temporal profile of protein expression in the uPA system was also investigated. UPARANT reduced both Müller cell gliosis and up‐regulated levels of inflammatory markers and exerted major anti‐apoptotic effects without influencing the autophagy cascade. Rescue from retinal cell degeneration was accompanied by improved retinal function. No scotopic phototransduction was rescued in the UPARANT‐treated animals as determined by the kinetic analysis of rod‐mediated a‐waves and confirmed by rod photoreceptor markers. In contrast, the cone photopic b‐wave was recovered and its rescue was confirmed in the whole mounts using cone arrestin antibody. Investigation of the uPA system regulation over RP progression revealed extremely low levels of uPA and its receptor uPAR both of which were recovered by HIF‐1α stabilization indicating that HIF‐1 regulates the expression of the uPA/uPAR gene in the retina. Ameliorative effects of UPARANT were likely to occur through an inhibitory action on up‐regulated activity of the αvβ3 integrin/Rac1 pathway that was suggested as a novel target for the development of therapeutic approaches against RP.

## 1 INTRODUCTION

Retinitis pigmentosa (RP) is a heterogeneous group of genetically inherited blinding disorders for which there are no treatments. It occurs in 1 out of 4000 people worldwide and is characterized by progressive photoreceptor loss. RP primarily affects the peripheral retina; it results from a genetic defect in rod photoreceptors and invariably evokes secondary cone photoreceptor loss that causes severe visual dysfunction. In addition to mutations in dozens of different genes, a chronic inflammation may be secondary to the primary genetic defect leading to rod death and gliotic events exacerbating inflammation thus establishing a positive feedback loop that subsequently strengthens retinal degeneration.^1^ The prominent role of inflammation in RP has been supported by several findings in humans and in animal models. For instance, the levels of inflammatory cytokines are higher in both the aqueous humour and the vitreous fluid of RP patients than in those of healthy controls.^2^ In addition, a recent study including RP patients treated with intravitreal dexamethasone has reported improved visual acuity in about half of the tested eyes.^3^ Moreover, in mouse models of RP, retinal levels of several inflammatory cytokines are increased^1^ while suppression of the gliotic response of Müller cells is effective in slowing down retinal degeneration.^4^ However, the complete knowledge of the events linking neuroinflammation to retinal degeneration is far to be reached.

Understanding the mechanism underlying inflammatory processes in RP is critical for development of sight‐saving therapeutics. In this respect, the urokinase‐type plasminogen activator (uPA) system seems to be a good candidate target to mediate inflammatory processes in RP. The uPA system consists in a group of proteases and protease inhibitors originally described for their role in regulating the activation of the zymogen plasminogen into its proteolytically active form, plasmin. However, the spectrum of action of the uPA system has been recently extended far beyond its classical pro‐angiogenic function and has emerged as a central actor in inflammatory processes.^5^ uPA is a serine protease that binds uPAR, a high affinity glycosyl‐phosphatidyl‐inositol‐anchored receptor that traditionally has been considered to focus proteolytic uPA activity on the cell membrane. However, uPAR also binds vitronectin and activates intracellular signalling through lateral interactions with its co‐receptors including integrins and the G‐protein‐coupled family of N‐formyl‐Met‐Leu‐Phe (fMLF) peptide receptors (FPRs). Both integrin receptors and FPRs seem to possess important regulatory effects in multiple pathological conditions, including inflammation.^6^, ^7^ Among integrin receptors, αvβ3 integrin is strongly implicated in regulatory functions mediated by the uPA system.^8^ In particular, αvβ3 integrin is coupled to inflammatory processes through the activation of integrin‐specific signalling pathways^9^, ^10^ among which an important role is played by Rac1, a small GTPase that seems to participate in the molecular machinery that is able to induce photoreceptor death in a mouse model of retinal degeneration.^11^

In the recent years, a major effort has been allocated to designing appropriate pharmacology for interfering with the uPA system. Among the inhibitors of the uPA system, the uPAR‐derived tetrapeptide Ac‐L‐Arg‐Glu‐L‐Arg‐L‐PheNH_2_, named RERF, has been designed to compete with fMLF for binding to FPRs although its low stability to the action of proteases was found to limit its dissemination.^12^ New N‐acetylated and C‐amidated peptide analogues containing α‐methyl α‐amino acids have been designed and synthesized to optimize the biochemical properties for therapeutic applications. Among these, Ac‐L‐Arg‐Aib‐L‐Arg‐L‐α(Me)Phe‐NH_2_, named UPARANT (recently designated as Cenupatide by the WHO's International Nonproprietary Names), adopts in solution a turned conformation similar to RERF, is stable in blood, displays a long‐time resistance to enzymatic proteolysis, competes with fMLF for binding to FPRs and may prevent integrin receptor activation without binding to uPAR or interfering with the uPA/uPAR binding.^13^ However, the precise mechanism of action of UPARANT remains to be fully elucidated. From a functional point of view, UPARANT has been initially characterized for its anti‐angiogenic properties using in vitro assays and animal models of proliferative ocular pathologies.^13^, ^14^, ^15^, ^16^, ^17^ More recently, an anti‐inflammatory activity of UPARANT has been deeply investigated using models of pathologies characterized by a negligible presence of angiogenic profiles.^18^, ^19^, ^20^, ^21^ Therefore, UPARANT seems to be a good candidate tool to unravel the inflammation cascade in RP.

In the present study, we addressed the anti‐inflammatory efficacy of UPARANT in the rd10 model of RP, one of the best models currently used to mimic the pathologic mechanisms of RP although some differences have been evidenced between mice and humans. This model is characterized by a delayed mutation in Pde6b (cGMP phosphodiesterase 6B, rod receptor, beta polypeptide), which results in photoreceptor degeneration that begins to be evident at about post‐natal day (PD) 15 and peaks at about PD20. In rd10 mice, UPARANT was administered systemically to determine its therapeutic value by evaluating its effect on gliotic response and inflammatory markers. Whether anti‐inflammatory action of UPARANT was coupled to neuroprotective activity was determined by assessing the levels of apoptotic and autophagic markers. In addition, UPARANT efficacy on dysfunctional electroretinogram (ERG) was also investigated with a particular focus on the relative contribution of rod and cone photoreceptors to partial ERG rescue. Finally, we measured protein levels in the uPA system over RP progression at the aim of evaluating which of the key players in the uPAR/co‐receptor pathway may participate to ameliorative effects of UPARANT.

## 2 MATERIALS AND METHODS

### 2.1 Experimental animals

C57BL/6J mice (used as wild‐type controls and from now on referred as control WT mice) and rd10 mutants (B6.CXB1‐Pde6brd10/J on a C57Bl6J background)^22^ were purchased from Charles River Laboratories, Italia (Calco, Italy) and mated in our breeding colonies. Experimental animals were housed in polycarbonate cages in groups of 4‐5 mice per cage (medium density) in a regulated environment (23 ± 1°C, 50 ± 5% humidity) with a 12 hours light/dark schedule (lights on at 08:00 am) and provided with a standard diet and water ad libitum. In rd10 mice, the presence of the homozygous Pde6b mutation was assessed periodically with PCR on DNA extracted from tail tissue.

Overall, 192 mice (60 WT and 132 rd10), either male or female, were used. Of the WT mice, 24 were used as controls. Of the rd10 mice, 24 mice for each experimental condition were either untreated, treated daily with PBS or with UPARANT to assess its efficacy in RP rescue. In each experimental group, 12 mice were randomly chosen and had electroretinography at PD30. After being killed, mice were arbitrarily subdivided into smaller groups for each of the other outcome measures. Additional 24 WT and 24 rd10 mice were used in experiments aimed at evaluating protein levels in the uPA system at both PD10 and PD15 (12 mice for each age in each group). Of the remaining mice, 12 WT and 36 rd10 were used in experiments aimed at investigating the role of hypoxia‐inducible factor 1 (HIF‐1) in the regulation of uPA/uPAR expression. Of them, 6 WT and 18 rd10 mice were grown in normoxia while 6 WT and 18 rd10 mice underwent to the oxygen‐induced retinopathy (OIR) protocol to determine HIF‐1 regulation of uPA/uPAR expression in hypoxic environment. All WT mice were left untreated. Out of the 36 rd10 mice, either normoxic or OIR, 12 mice were intravitreally injected with dimethyloxalylglycine (DMOG; see below) to stabilize the α subunit of HIF‐1 (HIF‐1α), while 12 mice received DMOG vehicle and 12 mice were left untreated. In all experiments, mice were anaesthetized using isoflurane before killing and then humanely killed by cervical dislocation.

Animal studies were carried out in compliance with the recommendations in the Guide for the Care and Use of Laboratory Animals of the National Institutes of Health, the ARVO Statement for the Use of Animals in Ophthalmic and Vision Research, the Italian guidelines for animal care (DL 6/14) and the European Communities Council Directive (2010/63/UE). The experimental procedures were approved by the Ethical Committee in Animal Experiments of the University of Pisa. All efforts were made to reduce animal suffering and the number of animals required to obtain reliable results was based on the rule of the replacement, refinement and reduction (the 3Rs).

### 2.2 Treatment with UPARANT

UPARANT was synthesized as succinate salt.^18^ UPARANT was dissolved in PBS. Treatments with UPARANT or PBS were initiated at PD10 and continued daily until PD30. Mice received UPARANT at 16 mg/kg via subcutaneous injection. This dose was based on previous findings obtained in our laboratory. In rats, for instance, subcutaneously administered UPARANT at 20 mg/kg was found to effectively reach the retina,^19^ whereas in either streptozotocin (STZ)‐treated rats or spontaneously diabetic Torii rats, UPARANT at 8 mg/kg was shown to efficiently ameliorate the pathological signs of diabetic retinopathy.^19^, ^20^ In addition, UPARANT at 8 mg/kg was found to improve diabetic kidney lesion in STZ‐treated rats.^21^ Here, the UPARANT dose used in rats was translated to mice by using the body surface area normalization method for an allometric dose translation.^23^ In all experiments, no statistical difference was observed between untreated and PBS‐treated rd10 mice. Therefore, data from PBS‐treated mice are not shown in the Results section.

### 2.3 Intravitreal injection of DMOG

Dimethyloxalylglycine (Cayman Chemical, East Ellsworth, MI) is an inhibitor of prolyl hydroxylase, the enzyme responsible for the hydroxylation of HIF‐1α that allows the protein to be targeted for degradation.^24^ DMOG was dissolved in dimethyl sulphoxide, diluted in PBS and was used at 1 mmol/L in line with previous studies in the mouse retina.^25^ DMOG was intravitreally injected (1 µL in each eye) under isoflurane anaesthesia at both PD12 and PD15 using a microsyringe (NanoFil syringe; World Precision Instruments, Sarasota, FL).

### 2.4 Oxygen‐induced retinopathy model

The OIR mouse model was generated as previously described.^26^ Briefly, newborn mice at PD7 and their nursing mothers were exposed to 75% oxygen in a hyperoxic chamber for 5 days, after which they were returned to room air for 5 days before to be killed.

### 2.5 ERG Recordings

Retinal function was examined at PD30 with scotopic and photopic full‐field ERG. Mice were dark adapted overnight prior to ERG recordings and their manipulation was done under dim red light. Mice were anaesthetized by intraperitoneal injection of avertin (1.2% tribromoethanol and 2.4% amylene hydrate in distilled water, 0.02 mL/g body weight; Sigma‐Aldrich, St. Louis, MO) and bilateral pupil mydriasis was induced by applying in both eyes a topical drop of 0.5% atropine. A heating pad was used to keep the body temperature at 38°C. The electrophysiological signals were recorded through silver/silver chloride ring electrodes inserted under the lower eyelids. The cornea was intermittently irrigated with saline solution to prevent clouding of the ocular media. Electrodes in each eye were referred to a needle electrode inserted subcutaneously at the level of the corresponding frontal region. The ground electrode was inserted subcutaneously in the tail. The electrodes were connected to a two‐channel amplifier. The light stimulation device consisted of Ganzfeld stimulator (Biomedica Mangoni, Pisa, Italy), which ensures a homogeneous illumination anywhere in the retina. Responses were collected simultaneously from both eyes, amplified at 1000 gain and filtered with a bandpass of 0.2‐500 Hz before being digitized at 5 kHz rate with a data acquisition device (Biomedica Mangoni). Initially, the electrical recordings were taken without any stimulus in order to measure the background noise levels. The scotopic responses, which primarily reflect rod function, were evoked by flashes with intensities ranging from −3.40 to 1.00 log cd‐s/m^2^. For each light intensity, a series of ERG responses were averaged (from a number of 25 responses for the dimmest stimulus intensity to a number of 5 for the brightest stimulus) and the interval between light flashes was adjusted to appropriate times that allowed response recovering (from 5 seconds for the dimmest stimulus intensities to 20 seconds for the brightest stimulus). After the completion of scotopic stimulation, photopic, cone‐mediated responses were recorded following 10 minutes light adaptation and evoked by flash intensities ranging from 0.34 to 1.00 log cd‐s/m^2^ that were presented on a 1.5 log cd‐s/m^2^ rod‐saturating background light. At each intensity, 25 ERG responses with an interstimulus interval of 3 seconds were averaged. All ERG waveforms were analysed using a customized program (Biomedica Mangoni). The signals were filtered using a butterworth second order bandpass filter from 1 to 300 Hz and the signals averaged. In compliance with the International Society for Clinical Electrophysiology guidelines, the b‐wave amplitude was measured from the trough of the a‐wave to the peak of the b‐wave or, if no a‐wave was present, from the pre‐stimulus baseline. For the noise measurement, root mean square of noise amplitude was measured.

The kinetic analysis of rod‐mediated a‐waves in the ERG was performed following the models and protocols employed by Lamb and Pugh^27^ and modified by Hood and Birch.^28^ To analyse the rod function, we used the following equation (Lamb‐Pugh model) to fit a series of a‐waves at increasing intensities:

$$R\left(t\right)=\left\{1-\text{exp}\left[-1/2\cdot \varphi \cdot A\cdot {\left(t-t_{\text{eff}}\right)}^{2}\right]\right\}\cdot R_{\max}$$

where *R*(*t*) is the amplitude of the a‐wave measured at the time *t*, *φ* is the number of photoisomerizations per rod produced by the flash, *A* is the amplification factor, *t* is the time after flash onset, *t*_eff_ is the effective delay or the delay between the flash and the electrophysiological response and *R*_max_ is the saturated amplitude of the a‐wave. Because the outer photoreceptor segments are shorter in rd10 mice,^29^ in this equation, *φ*, which is a value depending on the length of the outer photoreceptor, was substituted with *I* × *k* (in which *I* is the intensity of the flash in scotopic conditions and *k* is a variable depending on the number of photoisomerizations and the stimulus intensity).

### 2.6 Western blotting

For protein measurements, eyes were enucleated, the retinas were separated from the eyecups and stored at −80°C. Six samples were used for each experimental condition. Each sample contained two retinas from two different mice. Samples were lysed with RIPA lysis buffer (50 mmol/L Tris, pH 7.4 containing 150 mmol/L NaCl, 1% Triton X‐100, 1% sodium deoxycholate, 0.1% SDS, 5 mmol/L EDTA) and proteinase and phosphatase inhibitor cocktails (Roche Applied Science, Indianapolis, IN). Protein content was quantified by the Micro BCA Protein Assay (Thermo Fisher Scientific, Waltham, MA). Samples containing 30 µg of proteins were subjected to SDS‐PAGE (4%‐20%; Bio‐Rad Laboratories, Inc, Hercules, CA) and β‐actin was used as loading control. Gels were transblotted onto a PVDF membrane (Bio‐Rad Laboratories, Inc) and the blots were blocked in 3% skim‐milk for 1 hour at room temperature, followed by incubation overnight at 4°C with antibodies listed in Table 1. Blots were then incubated for 1 hour at room temperature with HRP‐conjugated secondary antibodies (1:5000) and developed with Clarity Western enhanced chemiluminescence substrate (Bio‐Rad Laboratories, Inc), images were acquired (ChemiDoc XRS^+^; Bio‐Rad Laboratories, Inc) and the optical density of the bands was evaluated (Image Lab 6.0 software; Bio‐Rad Laboratories, Inc). The data were normalized to β‐actin or to the total levels of proteins [for measurements of either the phosphorylated forms of signal transducer and activator of transcription (STAT) 3, cAMP response element‐binding protein (CREB), nuclear factor kappa‐light‐chain‐enhancer of activated B cells (NF‐κB) p65 or the Rac1 activity]. All experiments were performed in duplicate.

**Table 1:** Primary antibodies used in the Western blot analysis

Antibody	Dilution	Source	Catalogue
Mouse monoclonal anti‐GFAP	1:1000	Sigma‐Aldrich	G3893
Mouse monoclonal anti‐pSTAT3 (Tyr705)	1:200	Santa Cruz Biotechnology	sc‐8059
Rabbit polyclonal anti‐STAT3	1:200	Santa Cruz Biotechnology	sc‐482
Goat polyclonal anti‐pCREB (Ser133)	1:200	Santa Cruz Biotechnology	sc‐7978
Rabbit polyclonal anti‐CREB	1:200	Santa Cruz Biotechnology	sc‐25785
Rabbit polyclonal anti‐pNF‐kB p65 (Ser276)	1:200	Santa Cruz Biotechnology	sc‐101749
Rabbit polyclonal anti‐NF‐kB p65	1:200	Santa Cruz Biotechnology	sc‐372
Rabbit polyclonal anti‐iNOS	1:200	Santa Cruz Biotechnology	sc‐8310
Goat polyclonal anti‐ICAM‐1	1:200	Santa Cruz Biotechnology	sc‐1511
Rabbit polyclonal anti‐TNF‐α	1:1000	Abcam	ab6671
Rabbit polyclonal anti‐IL‐6	1:200	Abcam	ab6672
Rabbit polyclonal anti‐Bax	1:100	Santa Cruz Biotechnology	sc‐493
Rabbit polyclonal anti‐Bcl2	1:100	Santa Cruz Biotechnology	sc‐492
Rabbit monoclonal anti‐active caspase 3	1:1000	Cell Signaling Technology	9664
Rabbit polyclonal anti‐LC3	1:500	Cell Signaling Technology	4108
Rabbit polyclonal anti‐p62	1:200	Sigma‐Aldrich	P0068
Mouse monoclonal anti‐rhodopsin	1:2000	Abcam	ab5417
Rabbit polyclonal anti‐transducin α	1:1000	Abcam	ab74059
Rabbit polyclonal anti‐cone arrestin	1:500	Millipore	ab15282
Rabbit monoclonal anti‐uPA	1:1000	Abcam	ab133563
Rabbit polyclonal anti‐uPAR	1:500	Abcam	ab103791
Goat polyclonal anti‐FPR1	1:200	Santa Cruz Biotechnology	sc‐13198
Rabbit polyclonal anti‐FPR2	1:200	Santa Cruz Biotechnology	sc‐66901
Rabbit polyclonal anti‐FPR3	1:200	Santa Cruz Biotechnology	sc‐66899
Rabbit polyclonal anti‐pβ3 integrin (Tyr773)	1:500	Abcam	ab38460
Rabbit monoclonal anti‐αvβ3 integrin	1:1000	Novus Biologicals	NBP2‐67557
Mouse monoclonal anti‐Rac‐1	1:1000	Sigma‐Aldrich	05‐389
Rabbit polyclonal anti‐HIF‐1α	1:200	Santa Cruz Biotechnology	sc‐10790
Rabbit polyclonal anti‐VEGF	1:200	Santa Cruz Biotechnology	sc‐507
Mouse monoclonal anti‐β‐actin	1:25000	Sigma‐Aldrich	A2228

CREB, cAMP response element‐binding protein; FPR, formyl peptide receptors; GFAP, Glial fibrillary acidic protein; HIF‐1, hypoxia‐inducible factor 1; ICAM, intercellular adhesion molecule; IL, interleukin; iNOS, inducible nitric oxide synthase; NF‐kB, nuclear factor kappa‐light‐chain‐enhancer of activated B cells; STAT3, signal transducer and activator of transcription 3; TNF, tumour necrosis factor; uPA, urokinase‐type plasminogen activator; uPAR, uPA receptor; VEGF, vascular endothelial growth factor.

### 2.7 Immunohistochemistry and quantitative analysis

Cone photoreceptor immunohistochemistry and quantitative analysis were performed in retinal whole mounts of either WT or rd10 mice (6 retinas from untreated, vehicle‐or UPARANT‐treated mice, respectively). Anaesthetized mice were transcardially perfused with cold 4% paraformaldehyde in 0.1 mol/L phosphate buffer (PB), pH 7.4. The eyes were enucleated and the retinas were separated from the eyecups. The retinas were post‐fixed for 1.5 h at 4°C, rinsed in PB, immersed in sucrose (25% in PB) and stored at 4°C. Retinal whole mounts were rinsed in 0.1 mol/L PB and incubated for 1 hour at room temperature in blocking buffer (0.1 mol/L PB containing 10% donkey serum and 0.5% Triton X‐100) to prevent non‐specific labelling. Retinal whole mounts were then incubated for 72 hours at 4°C in primary rabbit polyclonal anti‐mouse cone arrestin antibody (1:500; Millipore, Bedford, MA, cat. AB 15282) diluted in 0.5% Triton X‐100‐containing 0.1 mol/L PB. After incubation, the whole mounts were rinsed in 0.1 mol/L PB and incubated for 48 hours at 4°C in Alexa Fluor 564 (1:200; Molecular Probes, Eugene, OR, cat. A‐11003) in 0.1 mol/L PB. Finally, the whole mounts were rinsed in 0.1 mol/L PB, mounted on gelatin‐coated glass slides photoreceptor side up and cover‐slipped with a 0.1 mol/L PB‐glycerin mixture. Immunostaining was viewed with a digital fluorescence microscope (Ni‐E; Nikon‐Europe, Amsterdam, The Netherlands) and immunofluorescent images were acquired using a digital camera (DS‐Fi1c; Nikon‐Europe). Electronic images were processed using an image‐editing software (NIS‐Elements software; Nikon‐Europe). The extent of the retinal area was measured (in pixels) using the freehand selection tool of an image‐editing software (ImageJ; http://imagej.nih.gov/ij/; provided in the public domain by the National Institutes of Health, Bethesda, MD). Counts of cone arrestin‐positive cells were averaged across the entire retina to give an overall cone density for each retina. In particular, cone density was measured by counting cones on images (125 × 125 µm each) taken at 20 retinal regions along the dorso‐ventral and naso‐temporal axis. Cones were counted on these images using the NIS‐Elements software (Nikon‐Europe). The total number of cones in the retina was obtained by multiplying the averaged local density by the corresponding retinal area.

### 2.8 Rac1 activity assay and immunoblotting

Rac1 activity was assessed using the Rac1 Activation Assay Kit (Sigma‐Aldrich). Six samples were used for each experimental condition. Each sample contained two retinas from two different mice. Samples were lysed using the lysis buffer provided in the kit. Three hundred micrograms of proteins was incubated with 5 µg of the Rac1/Cdc42‐binding domain of p21‐activated kinase protein bound to agarose beads for 1 hour at 4°C. The Rac1/Cdc42‐binding domain of p21‐activated kinase protein selectively recognizes the GTP bound (active) form of Rac1. The beads were then washed with the provided wash buffer followed by brief boiling in 2x sample buffer to release captured active Rac1‐GTP. Active Rac1‐GTP and the whole lysate were subjected to electrophoresis and analysed by immunoblotting as described above.

### 2.9 Data analysis

For the statistical analysis, Graph Pad Prism 5.03 was used (GraphPad Software, Inc, San Diego, CA). All data are expressed as means ± SEM and were analysed by the Shapiro‐Wilk test to certify normal distribution. One‐way ANOVA and post‐hoc analysis by Newman‐Keuls Multiple Comparison test were used for evaluating differences in protein levels as determined by Western blot. The same statistical analysis was used for evaluating the density of cone arrestin immunostaining. Two‐way ANOVA and post‐hoc analysis by Bonferroni's multiple comparison test were used for analysing ERG data. Differences with *P* < 0.05 were considered significant.

## 3 RESULTS

### 3.1 Effect of UPARANT on major pro‐inflammatory components of RP

**Figure 1:**
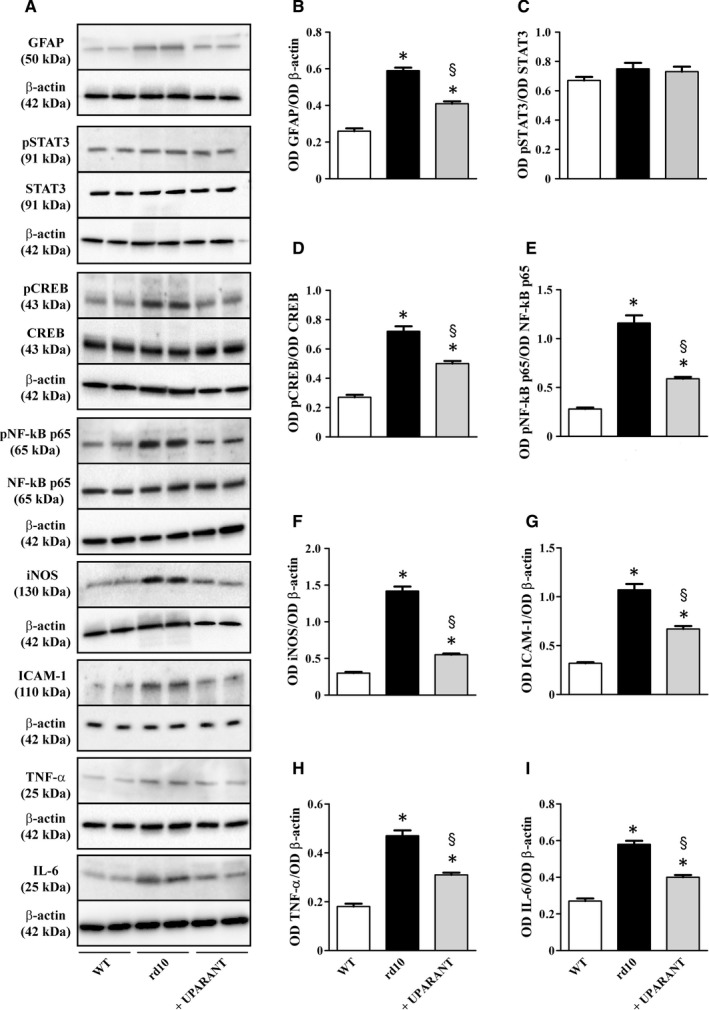
Effects of UPARANT on gliotic response and pro‐inflammatory markers. (A), Representative blots showing protein levels of glial fibrillary acidic protein (GFAP), phosphorylated signal transducer and activator of transcription (STAT3) at Tyr205, STAT3, phosphorylated cAMP response element‐binding protein (CREB) at Ser133, CREB, phosphorylated nuclear factor kappa‐light‐chain‐enhancer of activated B cells (NF‐kB) p65 at Ser276, NF‐*κ*B p65, inducible nitric oxide synthase (iNOS), intercellular adhesion molecule (ICAM)1, tumour necrosis factor (TNF)‐α and interleukin (IL)‐6 as evaluated by Western blot analysis in retinal extracts at PD30. β‐Actin was used as the loading control. (B‐I), Densitometric analysis showing that the levels of GFAP, phosphorylated CREB at Ser133, phosphorylated NF‐*κ*B p65 at Ser276, iNOS, ICAM‐1, TNF‐α and IL‐6 were higher in rd10 than in WT mice and were significantly reduced by UPARANT. No major differences between WT and rd10 mice were found for phosphorylated STAT3 (*P < 0.001 vs WT; ^§^ *P* < 0.001 vs rd10 untreated; One‐way ANOVA followed by Newman‐Keuls' multiple comparison post‐test). Data are mean ± SEM of values. Each histogram represents the mean ± SEM of data from six independent samples

Müller radial glial cells become activated in response to inflammatory stress as characterized by increased levels of glial fibrillary acidic protein (GFAP), an intermediate filament protein that is a very sensitive early indicator of retinal stress in Müller cells and is commonly used to characterize models of retinal degeneration including RP.^30^ As shown by the representative blots in Figure 1A (uncropped blots are shown in Figure S1) and the densitometric analysis of Figure 1B‐I, the levels of GFAP, the phosphorylation of both CREB at Ser133 and NF‐κB p65 at Ser276 and the levels of the inducible form of nitric oxide synthase (iNOS), intercellular adhesion molecule (ICAM)‐1, tumour necrosis factor (TNF)‐α and interleukin (IL)‐6 were higher in rd10 mice than in control WT mice. UPARANT reduced GFAP levels, the phosphorylation of both CREB and NF‐κB p65 as well as the levels of iNOS, ICAM‐1, TNF‐α and IL‐6 by approximately 1.4‐, 1.5‐, 2.2‐, 2.6‐, 1.6‐, 1.5‐ and 1.4‐fold, respectively (*P* < 0.001). No major differences were found for the phosphorylation of STAT3 at Tyr205.

### 3.2 Effects of UPARANT on retinal cell death: apoptosis and autophagy

**Figure 2:**
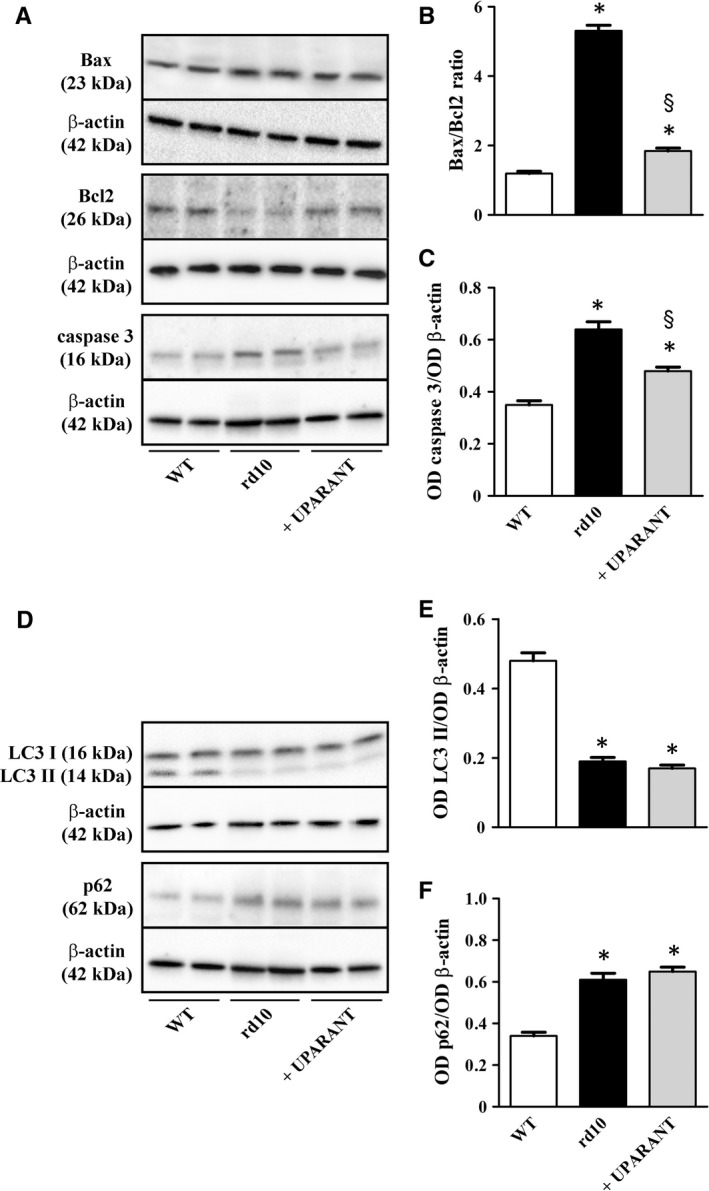
Effects of UPARANT on apoptosis and autophagy markers. (A), Representative blots showing protein levels of Bax, Bcl2 and caspase 3 as evaluated by Western blot in retinal extracts at PD30. β‐Actin was used as the loading control. (B,C), Densitometric analysis showing that the level of the anti‐apoptotic protein Bcl2 was lower in rd10 than in WT mice thus determining a drastic increase in the Bax/Bcl2 ratio that finally leads to increased active caspase 3. UPARANT recovered the normal Bax/Bcl2 ratio and reduced the active caspase 3 up‐regulation. (D), Representative blots showing protein levels of LC3 I, the lipidated form of LC3 (LC3‐II) and the autophagy substrate p62 as evaluated by Western blot in retinal extracts at PD30. β‐actin was used as the loading control. (E,F), Densitometric analysis showing that the levels of LC3‐II were lower in rd10 than in WT mice, whereas the levels of p62 were higher. No effects of UPARANT on autophagy markers could be observed (*P < 0.001 vs WT; ^§^ *P* < 0.001 vs rd10 untreated; One‐way ANOVA followed by Newman‐Keuls' multiple comparison post‐test). Data are mean ± SEM of values. Each histogram represents the mean ± SEM of data from six independent samples

We investigated whether UPARANT might affect the activation of selected components in the apoptotic pathway that is known to be dysregulated in the rd10 model.^31^ A major checkpoint in the apoptotic pathway is the ratio of pro‐apoptotic (Bax) to anti‐apoptotic (Bcl2) members that determines mitochondrial membrane damage, thus releasing cytochrome c into the cytosol and leading to the activation of caspases, which initiates the intracellular execution of cell death. As shown by the representative blots in Figure 2A (uncropped blots are shown in Figure S1) and the densitometric analysis of Figure 2B,C, the level of anti‐apoptotic Bcl2 was higher in rd10 mice than in WT mice thus determining a drastic increase in both the Bax/Bcl2 ratio and the levels of active caspase 3. UPARANT was found to reduce the Bax/Bcl2 ratio and active caspase 3 levels by approximately 2.9‐ and 1.3‐fold, respectively (*P* < 0.001). In retinal cell degeneration, autophagy is a conserved cellular self‐degradation process that not only plays a major role by serving as cell survival mechanism, but also contributes to cell death.^32^ In rd10 mice, we analysed the lipidation of the autophagosomal marker LC3 and we found that levels of the lipidated form of LC3 (LC3 II) were decreased, whereas those of the autophagy substrate p62 were increased (representative blots in Figure 2D and uncropped blots in Figure S1). Densitometric analysis (Figure 2E,F) demonstrates that, in respect to control WT mice, in rd10 mice, LC3 II levels were decreased and p62 levels were increased by approximately 2.5‐ and 1.8‐fold, respectively (*P* < 0.001). No effects of UPARANT on autophagy markers could be observed.

### 3.3 Partial rescue of photoreceptor dysfunction: UPARANT recovers cones while does not affect rods

**Figure 3:**
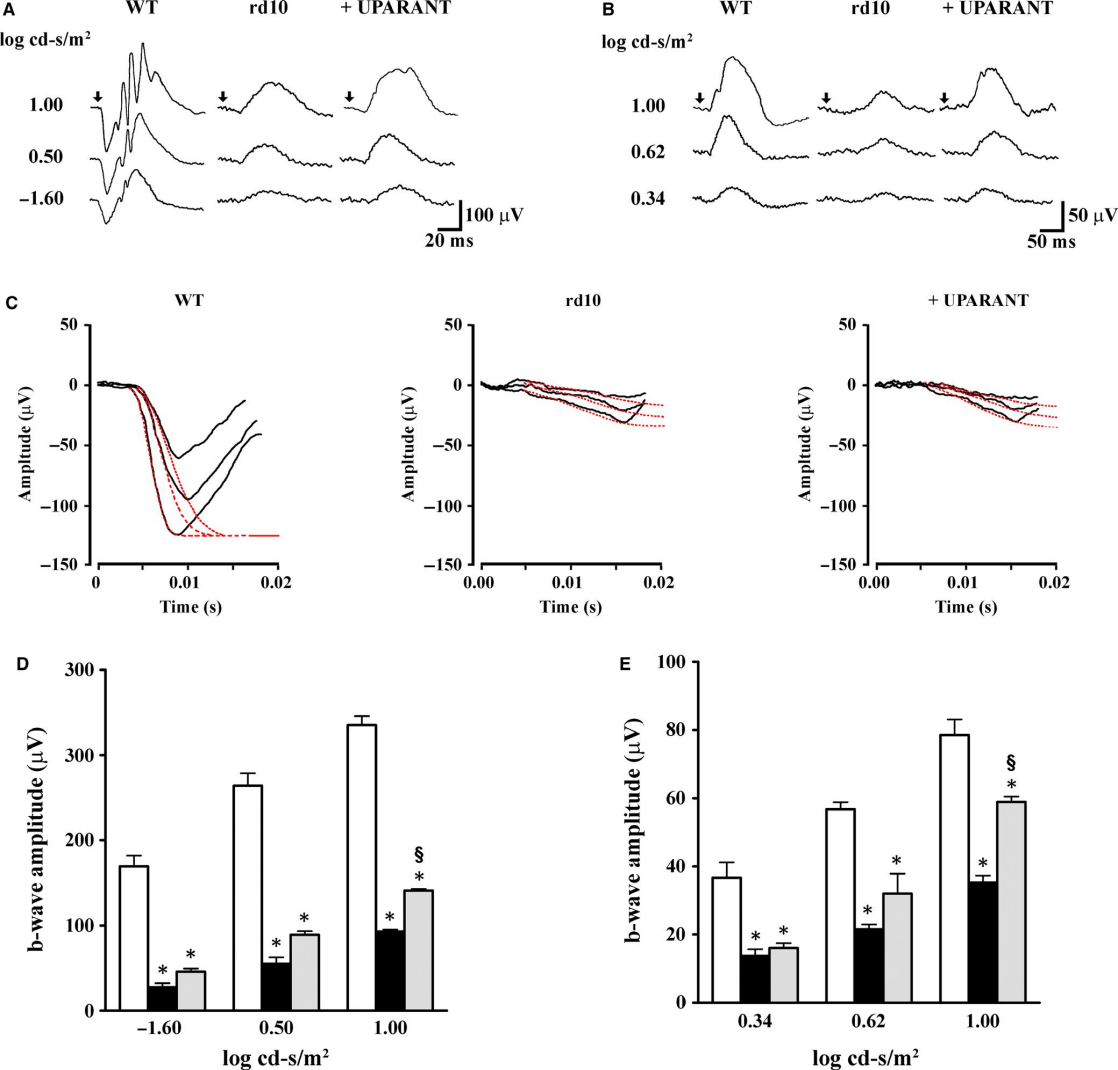
UPARANT‐induced improvement of the retinal light responses. (A,B), Representative electroretinogram traces recorded at PD30 in response to flashes with increasing intensities. Arrowheads indicate the onset of flash. (C), Representative a‐wave responses to flashes at −1.60, 0.50 and 1.00 log scotopic cd‐s/m^2^. The traces were fitted using the Lamb‐Pugh model (dotted lines) as described in Materials and Methods. The kinetic analysis of rod‐mediated a‐waves did not reveal significant differences between untreated and UPARANT‐treated rd10 mice. (D,E), Average peak amplitudes of scotopic and photopic b‐waves showing that the amplitudes of both scotopic and photopic b‐waves were smaller in rd10 than in WT mice and were significantly increased by UPARANT (*P < 0.001 vs WT; ^§^ *P* < 0.001 vs rd10 untreated; Two‐way ANOVA followed by Bonferroni's multiple comparison post‐test). Data are mean ± SEM of values. Each histogram represents the mean ± SEM of data from 12 independent samples. White bars: WT mice; black bars: untreated rd10 mice; grey bars: UPARANT‐treated rd10 mice

In additional experiments aimed at evaluating whether protective effects of UPARANT on apoptotic events were accompanied by ameliorated visual dysfunction, rd10 mice administered with UPARANT were subjected to comprehensive ERG analysis to assess rod and cone function. Representative ERG recordings are shown in Figure 3A (scotopic responses) and B (photopic responses). Based on ERG responses, small and variable a‐waves could be detected in rd10 mice without any difference between untreated and UPARANT‐treated animals. Additional kinetic analysis of rod‐mediated a‐waves allowed us to better delineate whether UPARANT might exert neuroprotective effects on rods. The results of this analysis are summarized in Figure 3C in which a series of a‐waves in response to increasing scotopic intensities were fitted using the Lamb‐Pugh model.^27^, ^28^ Table 2 shows that the amplification factor (*A*) was drastically reduced in rd10 mice in respect to WT mice, while the effective delay (*t*_eff_) was substantially increased. No significant differences were observed between untreated and UPARANT‐treated rd10 mice thus providing additional evidence that UPARANT did not improve rod photoreceptor function. Because a‐waves were variable and small and could not be measured reliably, we then chose b‐waves as an indirect measure of photoreceptor function. Quantitative analysis of the b‐wave amplitude is shown in Figure 3D,E. Responses from rd10 mice were compared with age‐matched WT mice. WT mice had robust photoreceptor responses, whereas in rd10 mice only weak photoreceptor responses could be detected. In contrast, rd10 mice treated with UPARANT had significantly improved amplitude of scotopic and photopic b‐waves. At 1.00 log cd‐s/m^2^, in rd10 mice treated with UPARANT, the amplitude of the scotopic and photopic b‐wave was about 42% and 75% of the corresponding amplitude in WT mice, respectively (*P* < 0.001).

**Table 2:** Electroretinogram a‐wave parameters

Parameter	WT	rd 10	+ UPARANT
A (s^‐2^)	9.3 ± 0.5	1.8 ± 0.2*	1.9 ± 0.2^a^
t_eff_ (ms)	5.1 ± 0.4	7.9 ± 0.6*	8.2 ± 0.9^a^

The amplification parameter (A) and the effective delay (t_eff_) were evaluated at a stimulus intensity of 1.00 log cd‐s/m^2^

* *P* < 0.01; One‐way ANOVA followed by Newman‐Keuls' multiple comparison post‐test). Data are mean ± SEM of values.

**Figure 4:**
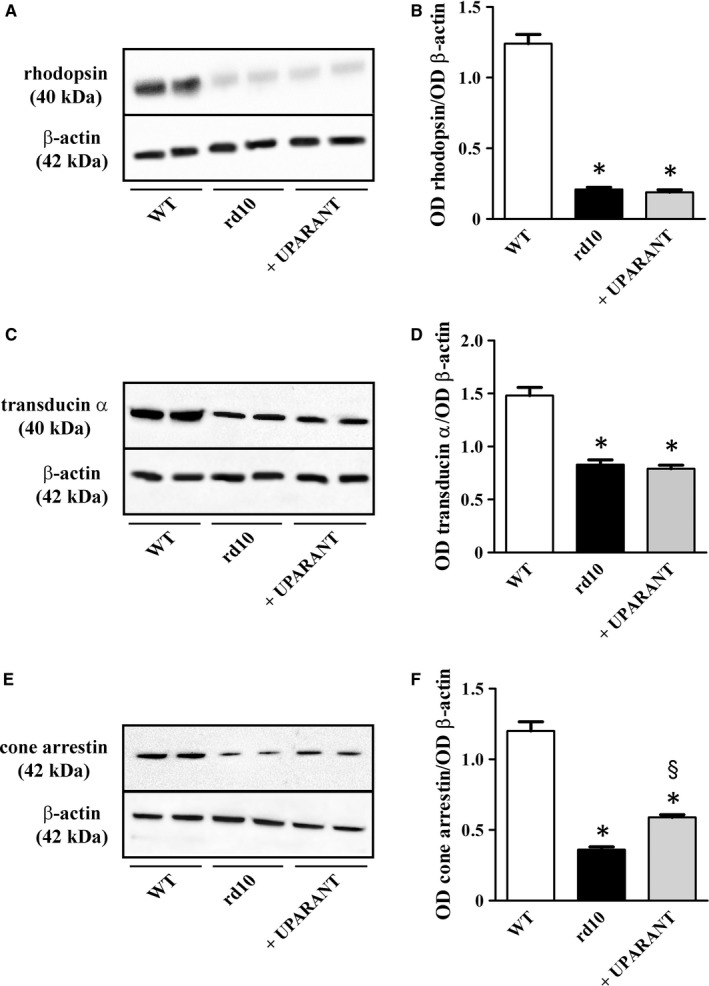
Effects of UPARANT on rod and cone markers. (A,C,E), Representative blots showing protein levels of rhodopsin (A), transducin α (C) and cone arrestin (E) as evaluated by Western blot in retinal extracts at PD30. β‐Actin was used as the loading control. (B,D,F), Densitometric analysis showing that the levels rhodopsin (B), transducin α (D) and cone arrestin (F) were lower in rd10 than in WT mice. UPARANT did not affect rhodopsin and transducin α while increased cone arrestin (*P < 0.001 vs control WT; ^§^ *P* < 0.001 vs rd10 untreated; One‐way ANOVA followed by Newman‐Keuls’ multiple comparison post‐test). Data are mean ± SEM of values. Each histogram represents the mean ± SEM of data from six independent samples

**Figure 5:**
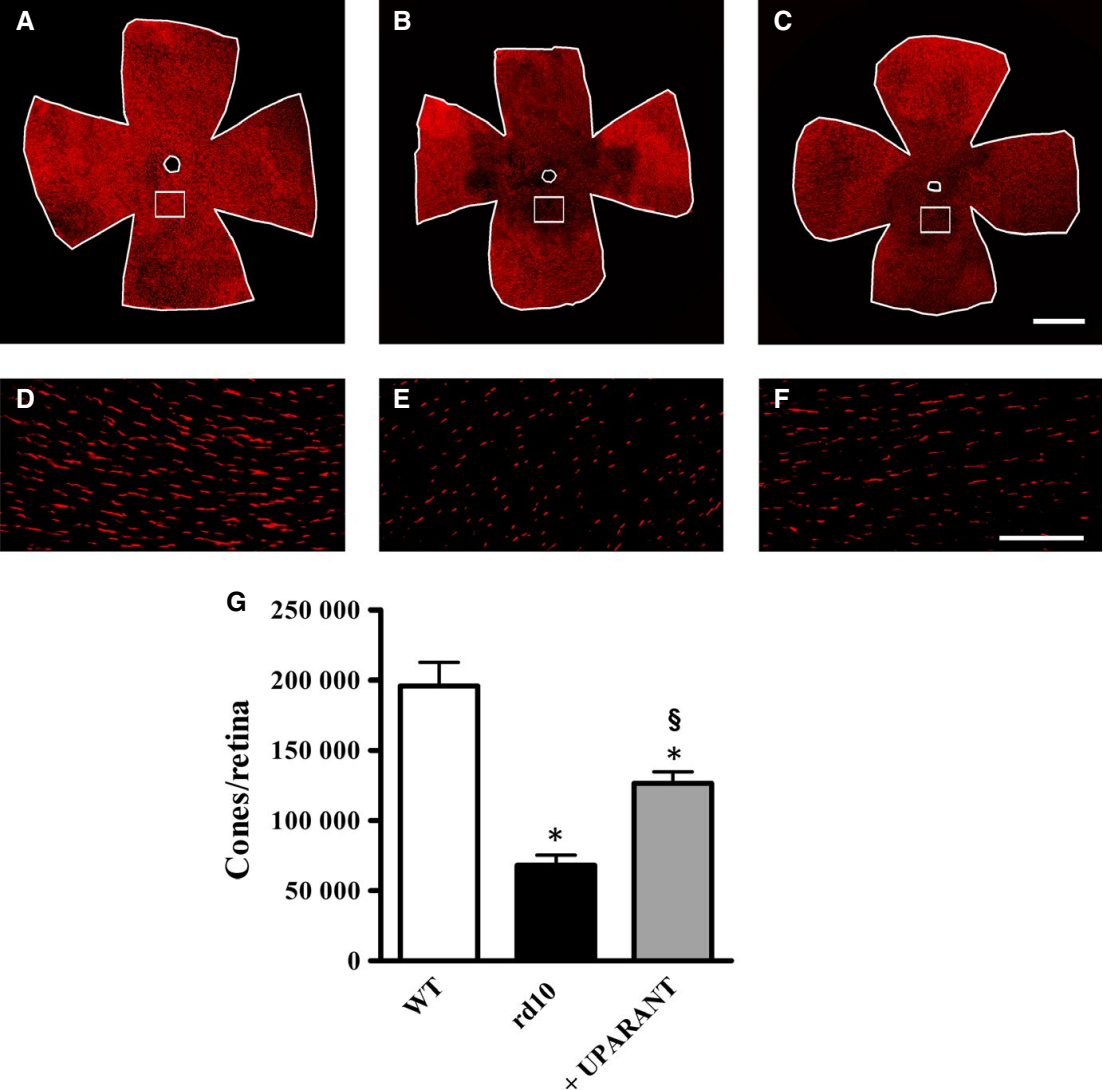
UPARANT‐induced recovery of cones. (A‐F), Fluorescence microscopy of cone arrestin immunoreactivity in retinal whole mounts and high magnification view of the boxed areas in the central retina demonstrates that in respect to WT mice (A,D) immunostaining for cone arrestin was visibly reduced in rd10 mice (B,E) whereas it was apparently increased after UPARANT treatment (C,F). (G), Counting of cone arrestin‐immunolabelled cells per retina demonstrates that rd10 mice treated with UPARANT had almost twice the cone density than untreated rd10 mice (*P < 0.001 vs WT; ^§^ *P* < 0.001 vs rd10 untreated; One‐way ANOVA followed by Newman‐Keuls’ multiple comparison post‐test). Data are mean ± SEM of values. Each histogram represents the mean ± SEM of data from six retinas for each experimental condition. Scale bars: 1 mm (A‐C) or 150 µm (D‐F)

The electrophysiological finding that no scotopic phototransduction was rescued in the UPARANT‐treated animals was confirmed by Western blot analysis of the rod markers rhodopsin and transducin α. The representative blots are shown in Figure 4A,C (uncropped blots are shown in Figure S1). The densitometric analysis of Figure 4B,D demonstrates that, in respect to WT mice, in rd10 mice, rhodopsin and transducin α levels were decreased by about 5.9‐ and 1.8‐fold, respectively (*P* < 0.001). No effects of UPARANT on rod markers could be observed. In contrast, UPARANT‐induced improvement of cone‐mediated responses was supported by the partial recovery of cone arrestin, a specific cone‐related opsin that is essential in the cone visual transduction cascade.^33^ Cone arrestin down‐regulation is a hallmark of cone degeneration in rd10 mice.^34^ Representative blots are shown in Figure 4E (uncropped blots are shown in Figure S1). As shown by the densitometric analysis in Figure 4F, in rd10 mice, cone arrestin levels were decreased by about 3.3‐fold with respect to WT mice, while in UPARANT‐treated mice cone arrestin levels were recovered by about 1.6‐fold with respect to untreated mice (*P* < 0.001). As shown by retinal whole mounts immunostained for cone arrestin, with respect to WT mice (Figure 5A), cone arrestin immunoreactivity was reduced in rd10 mice (Figure 5B), but was partially recovered by UPARANT treatment (Figure 5C). This effect was particularly evident in the central retina in which cone degeneration is known to proceed much faster than in the peripheral regions.^35^ High magnification of boxed areas in the central retina shown in Figure 5D‐F demonstrates that staining for cone arrestin was visibly increased in retinas from UPARANT‐treated rd10 mice. Counts of cones across the entire retina (Figure 5G) demonstrate that cone density was reduced by about 2.9‐fold in rd10 mice in respect to WT mice (from 196,000 ± 16,800 to 68,200 ± 7,120 *P* < 0.001) and that UPARANT partially prevented this reduction. In particular, after UPARANT, cone density was about 1.9‐fold higher than in untreated rd10 mice (*P* < 0.001) although still about 1.5‐fold lower than in WT mice (*P* < 0.001).

### 3.4 uPA system regulation over RP progression

**Figure 6:**
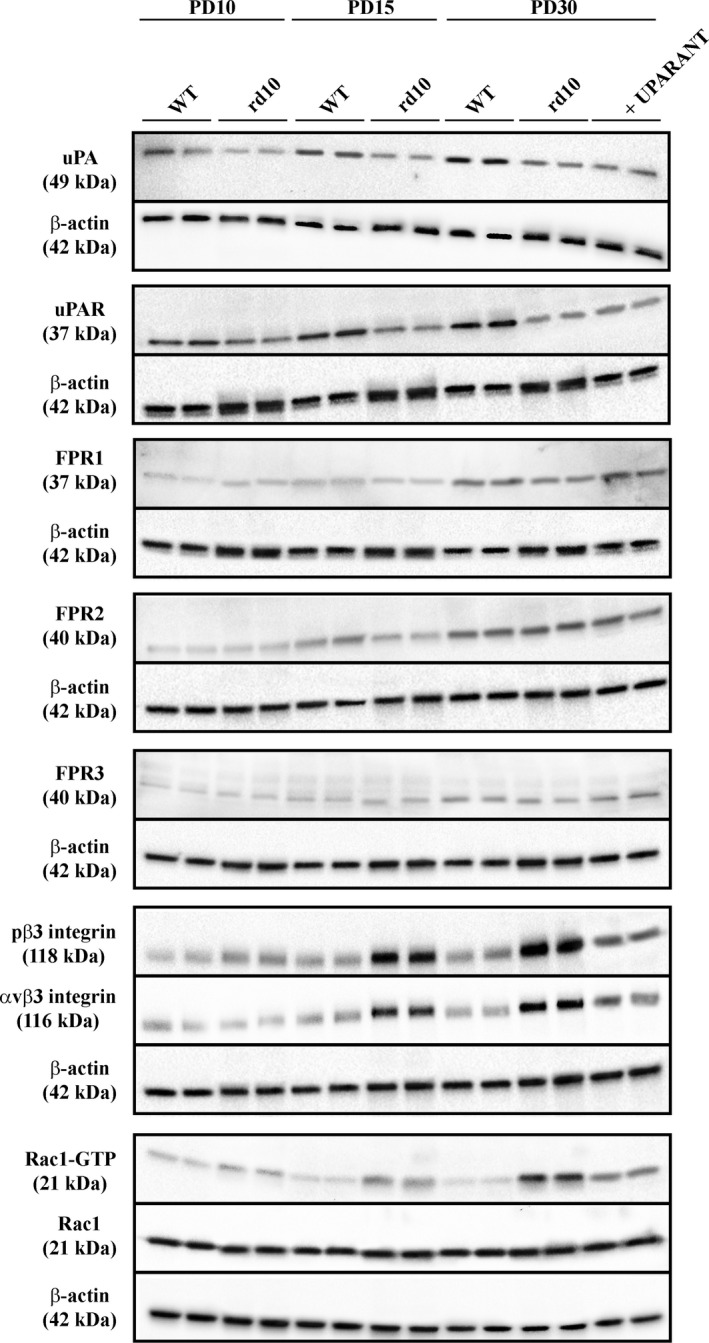
Protein expression in the urokinase‐type plasminogen activator (uPA) system over retinitis pigmentosa progression. Representative blots showing protein levels as evaluated by Western blot in retinal extracts at different post‐natal times. β‐Actin was used as the loading control

**Figure 7:**
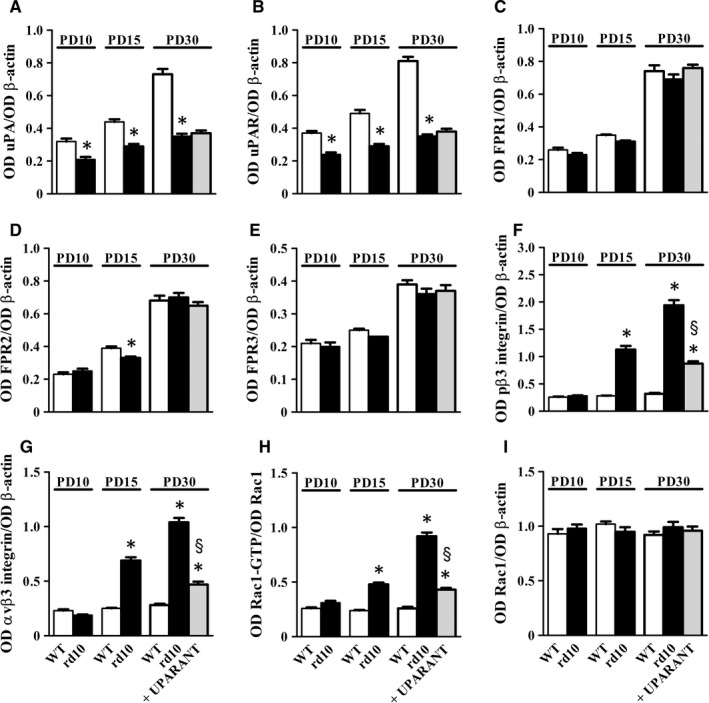
Effects of UPARANT on markers in the urokinase‐type plasminogen activator (uPA) system: inhibition of the αvβ3 integrin/Rac1 pathway. Protein levels as evaluated by the densitometric analysis of the blots represented in Figure 6. At any age, levels of uPA (A) and uPAR (B) in rd10 were lower than in WT mice. (C‐E), No differences were found for formyl peptide receptor (FPR) levels with the exception of FPR2 that at PD15 was lower in rd10 mice. (F‐H), Starting from PD15, the activity of αvβ3 integrin/Rac1 pathway was higher in rd10 mice in respect to WT mice. In fact, rd10 mice had higher levels of phosphorylated β3 integrin at Tyr773 (F) αvβ3 integrin (G) and Rac1 activity (H). No differences were found in Rac1 levels (I). UPARANT did not affect uPA, uPAR and FPRs while reduced phosphorylated β3 integrin and αvβ3 integrin levels. Rac1 activity as determined by Rac1‐GTP in respect to Rac1 levels was reduced by UPARANT (*P < 0.001 vs WT; ^§^ *P* < 0.001 vs rd10 untreated; One‐way ANOVA followed by Newman‐Keuls’ multiple comparison post‐test). Data are mean ± SEM of values. Each histogram represents the mean ± SEM of data from six independent samples

We investigated the temporal profile of protein expression in the uPA system. Representative blots of Figure 6 (uncropped blots are shown in Figure S1) are indicative of the protein levels in retinal extracts at different post‐natal times. Densitometric analysis is shown in Figure 7A‐I. In control WT mice, uPA expression was low at PD10 and significantly increased at PD15 without any further change between PD15 and PD30. Similar time course was observed for uPAR except for a significant amount of protein already present at PD10. FPR levels could be readily detectable at PD15, except for FPR1 whose expression was delayed to PD30. In general, FPR expression did not show significant changes over post‐natal development. At any age, both uPA and uPAR were significantly lower in rd10 mice with respect to WT mice. In particular, at PD10, PD15 and PD30, uPA levels in rd10 mice were approximately 1.5‐, 1.6‐ and 2.1‐fold lower than in WT mice, while uPAR levels were approximately 1.5‐, 1.7‐ and 2.3‐fold lower, respectively (*P* < 0.001). No differences in the expression of FPRs were observed between rd10 mice and WT mice with the exception of FPR2 that, at PD15, was about 1.2‐fold lower in rd10 mice than in control WT mice (*P* < 0.001). In the αvβ3 integrin pathway including its downstream effector Rac1, β3 integrin phosphorylation at Tyr773, αvβ3 integrin levels, Rac1 levels and Rac1 activity did not change over time in control WT mice. In rd10 mice, β3 integrin phosphorylation, αvβ3 integrin levels and Rac1 activity were higher than in WT mice at both PD15 (about 4.0‐, 2.8‐ and 2.0‐fold, respectively, *P* < 0.001) and PD30 (about 6.1‐, 3.7‐ and 3.5‐fold, respectively, *P* < 0.001). No appreciable differences over time were observed in Rac1 levels that did not differ between rd10 mice and WT mice at any age. UPARANT did not affect retinal levels of uPA, uPAR, FPRs and Rac1, whereas reduced the phosphorylation of β3 integrin, αvβ3 integrin levels and Rac1 activity by about 2.2‐, 2.3‐ and 2.1‐fold, respectively (*P* < 0.001).

### 3.5 HIF‐1 regulation of uPA/uPAR expression

**Figure 8:**
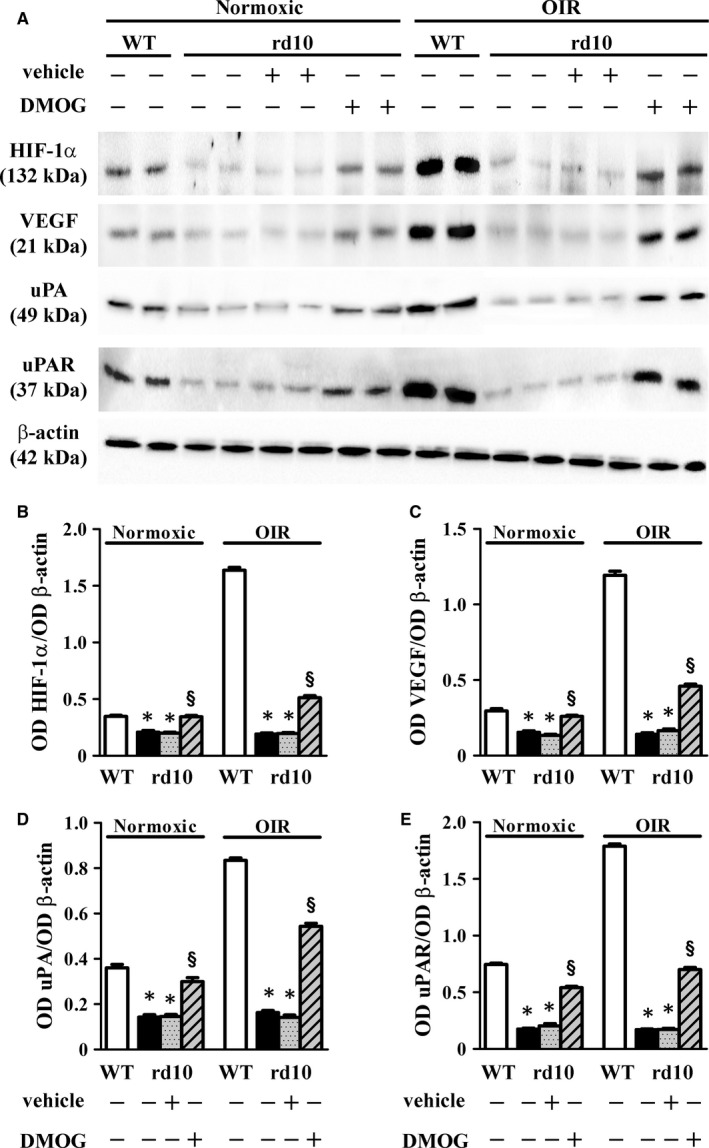
Effect of HIF‐1α stabilization on uPA/uPAR levels. (A), Representative blots showing protein levels of HIF‐1α, VEGF, uPA and uPAR as evaluated by Western blot in retinal extracts at PD17. β‐Actin was used as the loading control. (B‐E), Densitometric analysis showing that, in respect to WT mice, the levels of HIF‐1α, VEGF, uPA and uPAR were lower in rd10 mice either normoxic or subjected to the oxygen‐induced retinopathy (OIR) protocol. After HIF‐1α stabilization with intravitreal dimethyloxalylglycine (DMOG), the levels of HIF‐1α, VEGF, uPA and uPAR were higher than those in mice intravitreally injected with vehicle (*P < 0.001 vs respective WT; ^§^ *P* < 0.001 vs vehicle‐treated rd10; One‐way ANOVA followed by Newman‐Keuls’ multiple comparison post‐test). Data are mean ± SEM of values. Each histogram represents the mean ± SEM of data from 6 independent samples

We asked the question whether the extremely low levels of uPA/uPAR as determined in rd10 mice might depend on retinal expression of HIF‐1. In models of ocular pathologies characterized by HIF‐1 up‐regulation, uPAR is overexpressed in response to ischaemic conditions^15^ suggesting a causal relationship with HIF‐1 accumulation also in line with the finding that in tumour angiogenesis, HIF‐1 mediates the transcription of uPA/uPAR genes during hypoxia^36^, ^37^ In this respect, mouse models of retinal degeneration as the rd10 model are characterized by low levels of HIF‐1^38^, ^39^ thus allowing to check the possibility that restoring HIF‐1 by preventing the degradation of HIF‐1α, the oxygen‐sensitive subunit of HIF‐1, might recover retinal levels of uPA/uPAR. To this aim, we inhibited HIF‐1α degradation with intravitreal administration of DMOG and we found that HIF‐1α stabilization restored uPA and uPAR to levels similar to those in WT mice (Figure 8). Additional experiments with DMOG administration were also performed with use of the OIR model in rd10 mice. Indeed, mouse models of inherited retinal degeneration are unresponsive to hypoxia and lack of angiogenic profiles likely due to their low levels of both HIF‐1 and vascular endothelial growth factor (VEGF)^38^, ^39^ thus rendering them suitable to answer the question whether HIF‐1 regulates retinal expression of UPA/UPAR in hypoxic environment. As expected, in OIR rd10 mice, retinal levels of uPA and uPAR were drastically lower than in the OIR WT counterpart. Stabilization of HIF‐1α was able to recover at least in part retinal levels of HIF‐1/VEGF and to induce uPA/uPAR expression in response to hypoxia thus further supporting HIF‐1‐mediated regulation of uPA/uPAR in the retina. Blots in Figure 8A are representative of the protein levels of HIF‐1α, VEGF, uPA and uPAR in the different experimental conditions. Densitometric analysis shown in Figure 8B‐E demonstrates that in rd10 mice grown in normoxia used as controls, levels of HIF‐1α, VEGF, uPA and uPAR were lower than in WT mice (about 1.7‐, 1.4‐, 2.1‐ and 2.9‐fold, respectively, *P* < 0.001). In respect to vehicle treatment, DMOG treatment in rd10 mice caused increased levels of HIF‐1α, VEGF, uPA and uPAR (about 1.7‐, 2.0‐, 2.1‐ 2.0‐fold, respectively, *P* < 0.001). As expected, OIR WT mice exhibited up‐regulated levels of HIF‐1α, VEGF, uPA and uPAR that were drastically higher than those in OIR rd10 mice (about 8.5‐, 8.4‐, 5.1‐ and 10.5‐fold, respectively, *P* < 0.001). In respect to vehicle treatment, DMOG administration was found to increase the levels of HIF‐1α, VEGF, uPA and uPAR by about 2.6‐, 2.8‐, 3.8‐ and 4.1‐fold, respectively (*P* < 0.001) thus replacing at least in part levels measured in WT mice.

## 4 DISCUSSION

Increasing evidence is highlighting the critical role of the uPA system in inflammatory diseases and unchecked mechanisms underlying its function may represent hallmark diagnostic features of neuroinflammatory diseases of the retina. This study supports a role for the uPA system in RP pathophysiology through a mechanism presumably involving the activation of the αvβ3 integrin/Rac1 pathway by inflammatory molecules responsible for the chronic inflammation induced by the primary rod death. The activation of the αvβ3 integrin/Rac1 pathway would represent the first step in establishing a positive feedback loop that, by enhancing the inflammatory state of the retina, may actively contribute to the secondary cone death. This study also provides evidence that systemic administration of UPARANT may be an effective, non‐invasive approach to alleviate the pathological signs of RP.

### 4.1 UPARANT pharmacokinetics, delivery and efficacy

The use of the systemic route to deliver UPARANT to the eye can be traced back to 2016 when some experiments with subcutaneous administration of the drug were added to the main body of evidence demonstrating the efficacy of intravitreally injected UPARANT in counteracting choroidal neovascularization using the nAMD model.^15^ Systemic administration was used as a convenient and less invasive delivery route, particularly if considering long‐term use of a drug and the need of preserving the retina when repeated ERG recording is necessary to determine drug efficacy on visual dysfunction. On the other hand, systemic delivery to the posterior eye can be limited by the BRB that may prevent drug diffusion to the choroid and the retina. However, in the nAMD model, UPARANT systemically delivered at 40 mg/kg was shown to reduce laser‐induced VEGF up‐regulation similar to intravitreal injections at 4 µg/µL indicating that the drug was taken up by the tissue from the administration site and conveyed to the posterior segment of the eye by the blood flow^15^. This was confirmed by recent findings demonstrating that UPARANT, administered subcutaneously at 20 mg/kg, reaches rapidly the blood systemic circulation and remains detectable in the plasma until 24 hours^19^ suggesting a slow elimination rate of the compound in line with its high plasma stability.^13^ As shown by additional findings, UPARANT can be detected in the eye 2 hours after its administration and its delivery to the retina is approximately 15% of that in the eye indicating its ability to cross the BRB and supporting the validity of subcutaneous administration as potentially effective in the treatment of ocular diseases. After 24 hours, UPARANT level in the eye is about 20% of that at 2 hours indicating a half‐life of approximately 10‐11 hours and suggesting a potential long duration of the intraocular pharmacologic effect.^19^ UPARANT dose and regimen used here are in line with those used in previous studies in rats in which systemic treatment with the drug was shown both effective and safe.^19^, ^20^

### 4.2 The uPA system and inflammation

Here, we evaluated whether UPARANT, a peptide targeting the uPA system that is endowed with a marked anti‐inflammatory activity, may be effective in counteracting neuroinflammation in the rd10 mouse model of RP. Therapeutic strategies targeting inflammatory processes have been designed and applied to slow down the degenerative process as in patients with cystoid macular oedema secondary to RP in which promising potential of improving visual acuity by repeated intravitreal steroids or a sustained‐release dexamethasone implants highlights the predominant inflammatory reaction consequent to photoreceptor degeneration.^3^ In this respect, the identification of therapeutic approaches targeting inflammation is actively pursued by basic research given the potential side effects of the present anti‐inflammatory treatments and the insufficient clinical evidence to support their use in RP patients.

The uPA system provides an integrated multimolecular complex that exerts pleiotropic functions including an important role in angiogenesis and inflammation.^5^ In the diseased retina, the inhibition of the uPA system has been shown to block critical processes involved in both angiogenesis and inflammation,^14^, ^15^, ^19^, ^20^ but no information is available in the rd10 model of RP. As shown by the present findings, targeting the uPA system with UPARANT results in major anti‐inflammatory action including reduction of Müller cell gliosis, as characterized by decreased up‐regulation of both GFAP and pro‐inflammatory markers. In RP, as a consequence of gliotic processes, Müller cells release factors that exacerbate inflammation, establishing a positive feedback loop driving inflammatory processes.^1^ Among inflammatory factors, ICAMs are a family of type I transmembrane proteins that bind to integrins and play a central role in the activation of intracellular signalling pathways, inflammation and immune responses.^40^ The present finding that UPARANT reduces GFAP expression and dampens the production of inflammatory factors suggests that this compound may act by breaking the positive feedback loop between Müller cell gliosis and release of pro‐inflammatory factors thus resulting in a reduced inflammatory drive. In this respect, pharmacological strategies preventing gliotic responses of Müller cells have been proposed to slow down retinal degeneration in the rd10 model.^4^

### 4.3 Protective effects of UPARANT and rescue of visual impairment

Concomitantly with reducing inflammation, UPARANT exerts major neuroprotective effects that result in a partial restoring of visual impairment. The present findings demonstrate that UPARANT reduces the Bax/Bcl2 ratio by increasing Bcl2 levels without affecting Bax thus suggesting Bcl2 as a major target in UPARANT anti‐apoptotic action. Recovered Bcl2 results in reduced caspase 3 activation that finally preserves at least in part the retina from cell death. As shown by the present data, rd10 mice are also characterized by reduced lipidation of the autophagosomal marker LC3‐II and lower levels of autophagy regulators thus suggesting a marked reduction in autophagy flux in line with previous results also demonstrating that normalizing the autophagy flux is a good therapeutic option to treat RP.^41^ On the other hand, the role of autophagy in RP is controversial as moderate levels of autophagy may be beneficial while excessive autophagy may be deleterious.^42^ As shown here, ameliorative effects of UPARANT on retinal cell death do not involve an improved autophagic cascade suggesting that decreased apoptosis is sufficient per se to partially protect retinal cells from degeneration.

Reduced retinal cell death contributes to prevent visual impairment, thus supporting the notion that inflammation recovery results in photoreceptor rescue and ameliorated visual dysfunction.^43^ In the rd10 model, reduced photoreceptor input to bipolar cells that are known to generate the b‐wave together with Müller cells, determines reduced signal transduction from bipolar to amacrine cells. In fact, around PD30, there are only 24% remaining scotopic b‐wave amplitude and 44% remaining photopic b‐wave amplitude^34^ in line with the values determined here. As also shown here, UPARANT‐treated rd10 mice do not seem to display reliably measurable a‐waves, which in the mouse arise almost exclusively from the rod photoreceptors.^44^ Thus, no scotopic phototransduction is apparently rescued by drug administration. This is supported by the kinetic analysis of rod‐mediated a‐waves further indicating that UPARANT does not affect rod neurodegeneration as also confirmed by the lack of rod preservation using Western blot analysis of rod photoreceptor markers. On the other hand, improvement of scotopic b‐waves after UPARANT can be attributed to partially recovered post‐receptor signalling in the cone‐mediated pathway that is established when most of the rods are found to degenerate.^45^ While ineffective on rods, UPARANT exerts neuroprotective effects on cones as demonstrated here by the increased amplitude of cone photopic b‐waves and also supported by a significant preservation in cone arrestin loss and a substantial rescue of cones in the entire retina thus indicating that UPARANT differentially interferes with mechanisms underlying rod or cone death. As rods degenerate for a genetic defect and cones die more slowly due to inflammation, it is conceivable that UPARANT may have no effect on rod degeneration while mainly counteracting inflammatory‐driven cone death. In this respect, there is scarce evidence of rod protection from degeneration although some findings are indicative of partially rescued rod photoreceptor death that may participate to improve cone survival as for instance after treatments with anti‐inflammatory drugs.^46^, ^47^

### 4.4 Differential regulation in the uPA system

Evaluation of protein levels in the uPA system demonstrates that uPA and uPAR are drastically down‐regulated over RP progression. This is in apparent contrast with the fact that increased expression of the uPA system is generally coupled to inflammatory processes in the diseased retina.^20^ The finding that HIF‐1α stabilization almost recovers retinal levels of uPA and uPAR suggests a strict correlation between the activity of HIF‐1 and the expression of uPA/uPAR in the retina in line with the demonstration that uPA/uPAR gene transcription is mediated by HIF‐1 in models of tumour angiogenesis.^36^, ^37^ In this respect, RP models are characterized by low levels of HIF‐1 because of a concomitance of events including the less HIF‐1 expression by photoreceptors due to their loss^48^ as well as the decreased oxygen consumption by dying photoreceptors that generate a hyperoxic environment leading to HIF‐1α degradation.^49^ The additional evidence that HIF‐1α stabilization in the OIR model almost restores uPA/uPAR expression in response to hypoxia further supports HIF‐1‐mediated regulation of uPA/uPAR in the retina.

Of the uPAR co‐receptors, FPR levels are unaltered in rd10 mice although in the presence of a negligible amount of uPA/uPAR thus suggesting that FPRs may function independently on uPAR and possibly excluding their role in inflammatory events that characterize RP. On the other hand, the additional finding of an increased activity of the αvβ3 integrin/Rac1 pathway is indicative of integrin involvement in the inflammatory cascade triggered by photoreceptor degeneration. Of the beta subunits assembled into different integrin heterodimers in the integrin family of matrix receptors, αvβ3 integrin is the primary integrin heterodimer and is a key contributor to inflammation. In particular, there is evidence that the αvβ3 integrin pathway plays a role in promoting stress‐induced endothelial cell activation by regulating NF‐kB‐induced pro‐inflammatory responses.^9^ In addition, in cultured astrocytes, inflammation induces the expression of αvβ3 integrin that appears to regulate astrocyte reactivity.^50^ In models of diabetic nephropathy, up‐regulated levels of αvβ3 integrin and increased Rac‐1 activity are associated with main inflammatory processes as demonstrated by ameliorative effects of systemic UPARANT administration.^21^ In the diseased brain, activation of αvβ3 integrin promotes the release of inflammatory factors while its inhibition has been shown to reduce brain inflammatory reactions.^51^, ^52^ On the other hand, activation of the β3 integrin pathway seems to promote brain repair in the recovery phase from an ischaemic stroke.^40^

Information about αvβ3 integrin role in the diseased retina is limited. Much work has been performed in retinal models of neovascularization in which the αvβ3 integrin pathway is important for the activation of key receptors involved in neovessel formation.^53^ In rd10 mice, αvβ3 integrin blockade results in degeneration recovery through inhibition of microglial phagocytosis.^54^ In addition, reduced Rac1 activity results in protective effects on rod degeneration through a modulation of oxidative stress.^11^

As shown by the present results, UPARANT, a compound traditionally supposed to inhibit the interaction between uPAR and FPRs acts here by ameliorating the pathological signs of RP, although in the presence of negligible amount of uPAR and therefore resizing our interpretation on UPARANT mechanism of action. In this respect, UPARANT has been designed to mimic the sequence through which uPAR interacts with FPRs thus competing with FPR ligands. On the other hand, UPARANT may also directly bind to αvβ3 integrin that is indeed forced into an inactive state and it may prevent integrin receptor activation without binding to uPAR or interfering with the uPA/uPAR binding.^13^ In addition, in the absence of FPRs, the compound may bind to the cell surface at picomolar concentrations in an integrin‐dependent manner.^14^, ^55^ If one considers that multiple αvβ3 antagonists are currently undergoing clinical trials for the treatment of inflammation‐dependent diseases,^56^ then the possibility that UPARANT targets the αvβ3 integrin pathway should deserve further investigation.

## 5 CONCLUSION

**Figure 9:**
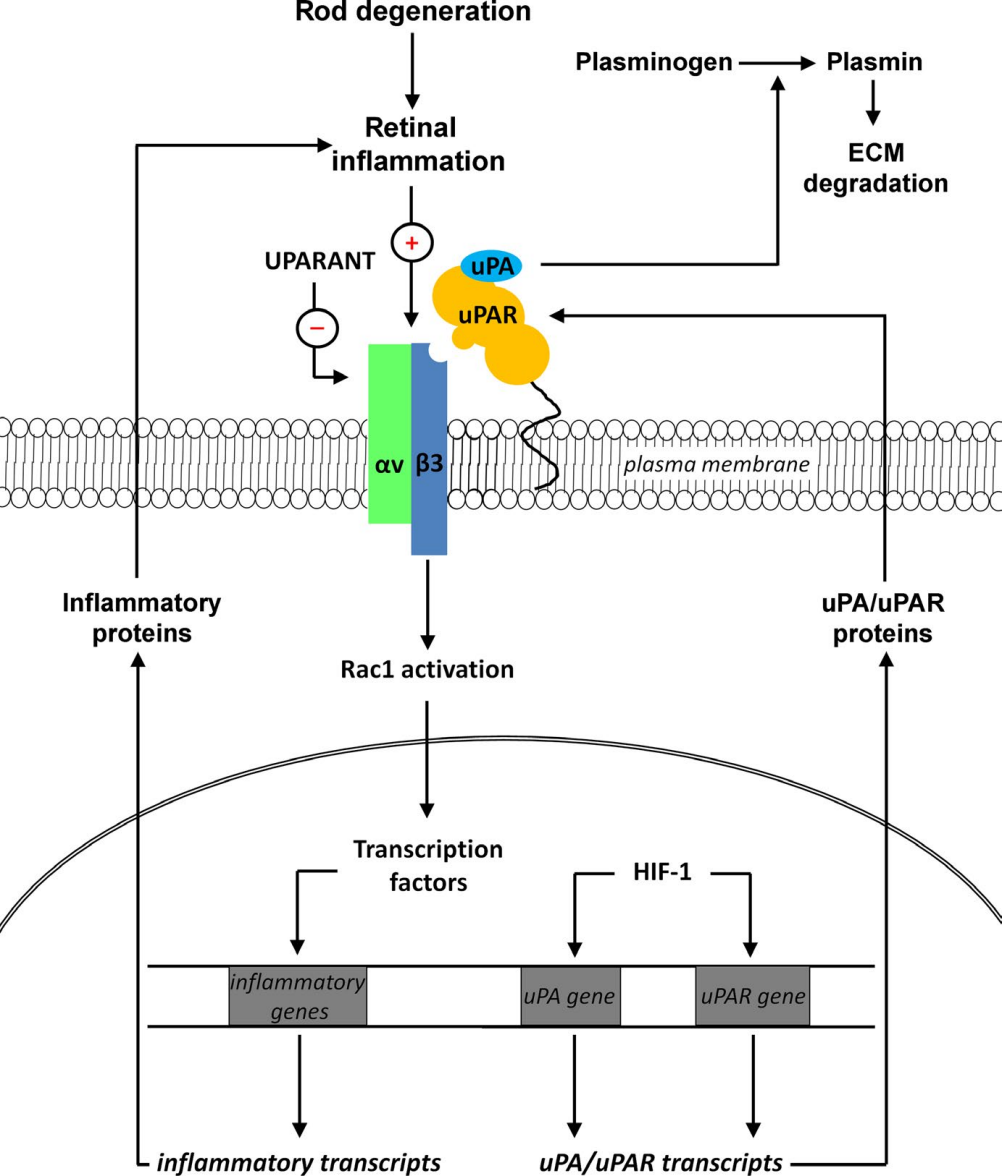
Hypothetical model of urokinase‐type plasminogen activator (uPA) system function in retinitis pigmentosa (RP). Upon binding to uPAR, uPA catalyses the conversion of plasminogen into plasmin, a serine protease involved in extracellular matrix (ECM) degradation and cell motility. As uPAR lacks an intracellular domain, its uPAR**_88‐92_** sequence forms supramolecular complexes by interacting with transmembrane receptors of which the αvβ3 integrin, originally named vitronectin receptor, acts through multiple intracellular signalling including Rac1 to regulate the transcription of different genes including those encoding inflammatory factors. In the rd10 model, low retinal levels of HIF‐1α may prevent uPA/uPAR transcription thus resulting in low levels of uPA/uPAR proteins that impede plasmin function. Inflammation consequent to rod degeneration may induce αvβ3 integrin expression thus presumably exacerbating inflammatory processes. UPARANT improves RP‐associated damages possibly through inhibition of up‐regulated levels of αvβ3 integrin/Rac1 pathway thus suggesting that the drug may act downstream uPAR. αv, αv integrin subunit; β3, β3 integrin subunit; HIF‐1, hypoxia‐inducible factor 1; uPAR, uPA receptor

Together, the present findings support the possibility that the uPA system may be coupled to retinal inflammation in RP and that inhibition of up‐regulated αvβ3 integrin/Rac1 pathway may attenuate photoreceptor cell loss through a major anti‐inflammatory action. Hypothetical model of the regulation of inflammatory processes by the uPA system is shown in Figure 9. Our hypothesis is that inflammation consequent to rod degeneration may induce αvβ3 integrin up‐regulation and that its inhibition by UPARANT may improve RP‐associated damages. In this respect, preventing cone loss is of particular importance as irreversible visual impairment is one of the most important clinical problems of RP and its possible treatments are limited by the scarce availability of drugs ameliorating visual dysfunction. Overall, the present findings add further evidence to the potential application of UPARANT although the extrapolation of these experimental findings from the rd10 mouse model to the clinic is not straightforward.

## CONFLICT OF INTEREST

MC received a grant from Kaleyde Pharmaceuticals AG. MDR. and Vi.P. are the holders of UPARANT patent. MC, MDM., FP, Va.P., and PB declare no conflicts of interest.

## AUTHORS’ CONTRIBUTION

PB, MDM. and Vi.P. designed the experiments and interpreted the results. MC designed and interpreted the electrophysiological study. MC, MDM, FL and Va.P. performed the experiments and analysed the data. PB and MDM. wrote the paper. MDR. and Vi.P. contributed to the final revision of the paper. All authors approved the final manuscript.

## Data Availability

The data that support the findings of this study are available from the corresponding author upon reasonable request.
