# Supplementary material for: The urokinase‐type plasminogen activator system as drug target in retinitis pigmentosa: New pre‐clinical evidence in the rd10 mouse model
Source: J Cell Mol Med. 2019 Jun 28;23(8):5176–92. doi: 10.1111/jcmm.14391 (PMC6653070; doi:10.1111/jcmm.14391)

Supporting Figure S1: uncropped Western blots

GFAP/β-actin (Fig. 1A)

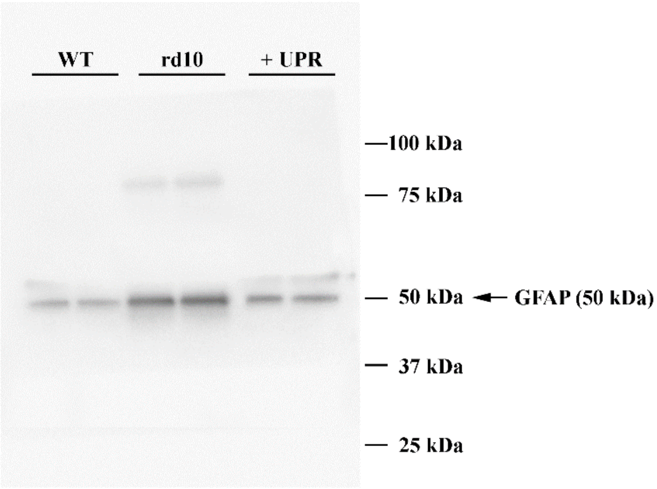

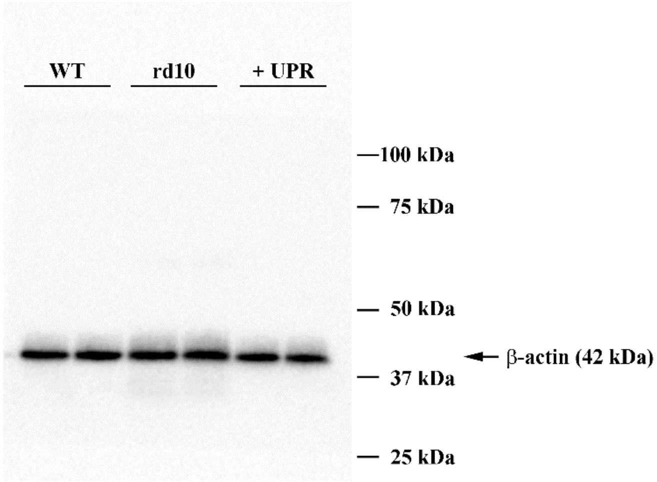

pSTAT3/STAT3/β-actin (Fig. 1A)

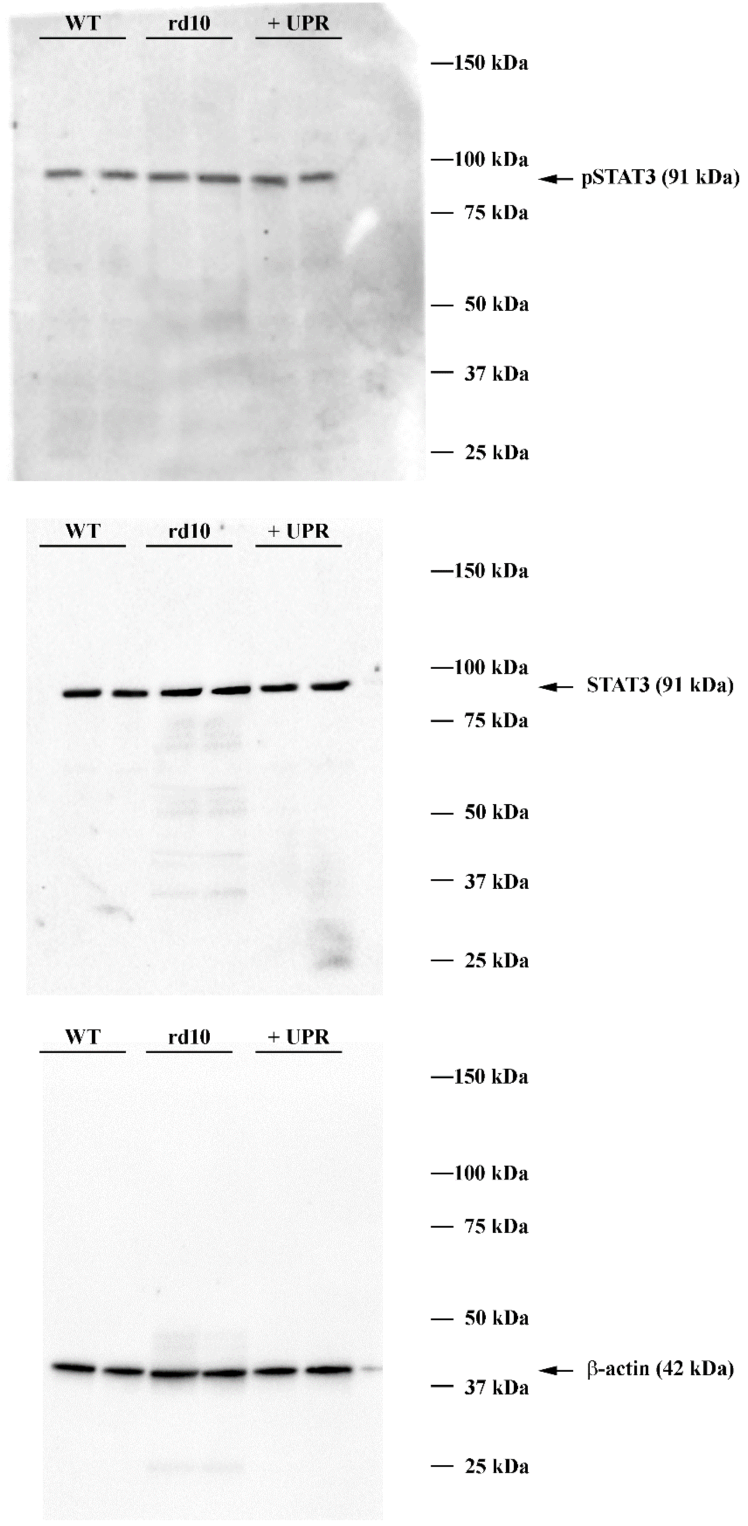

pCREB/CREB/β-actin (Fig. 1A)

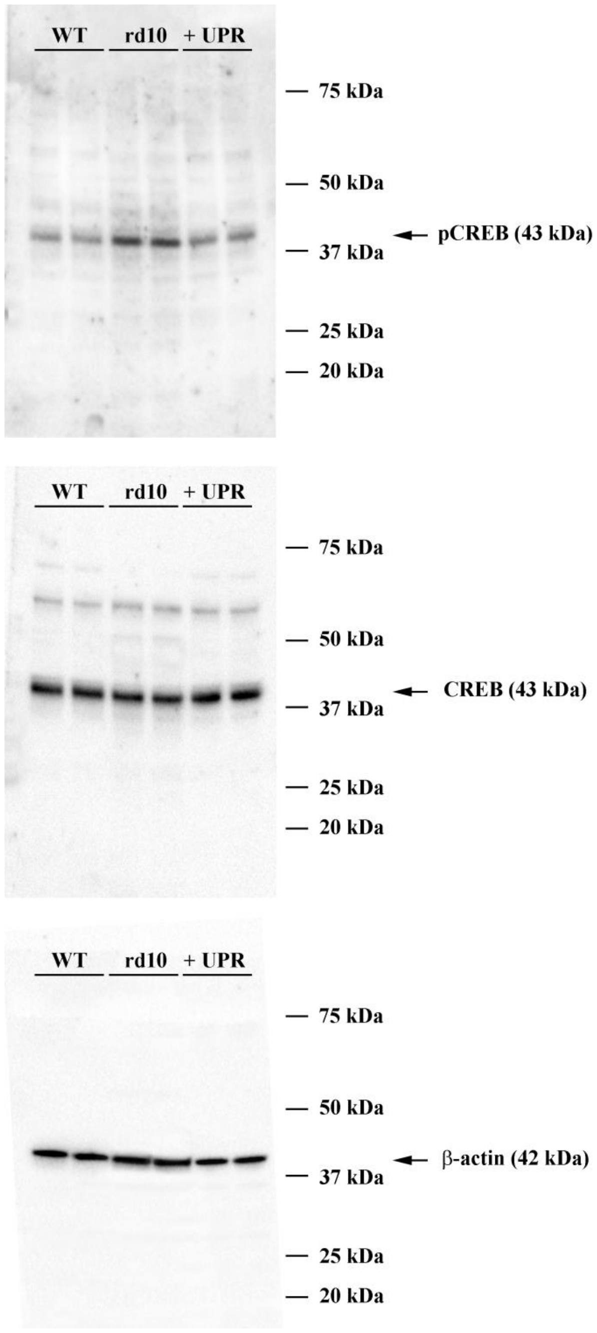

pNFkB p65/NFkB p65/β-actin (Fig. 1A)

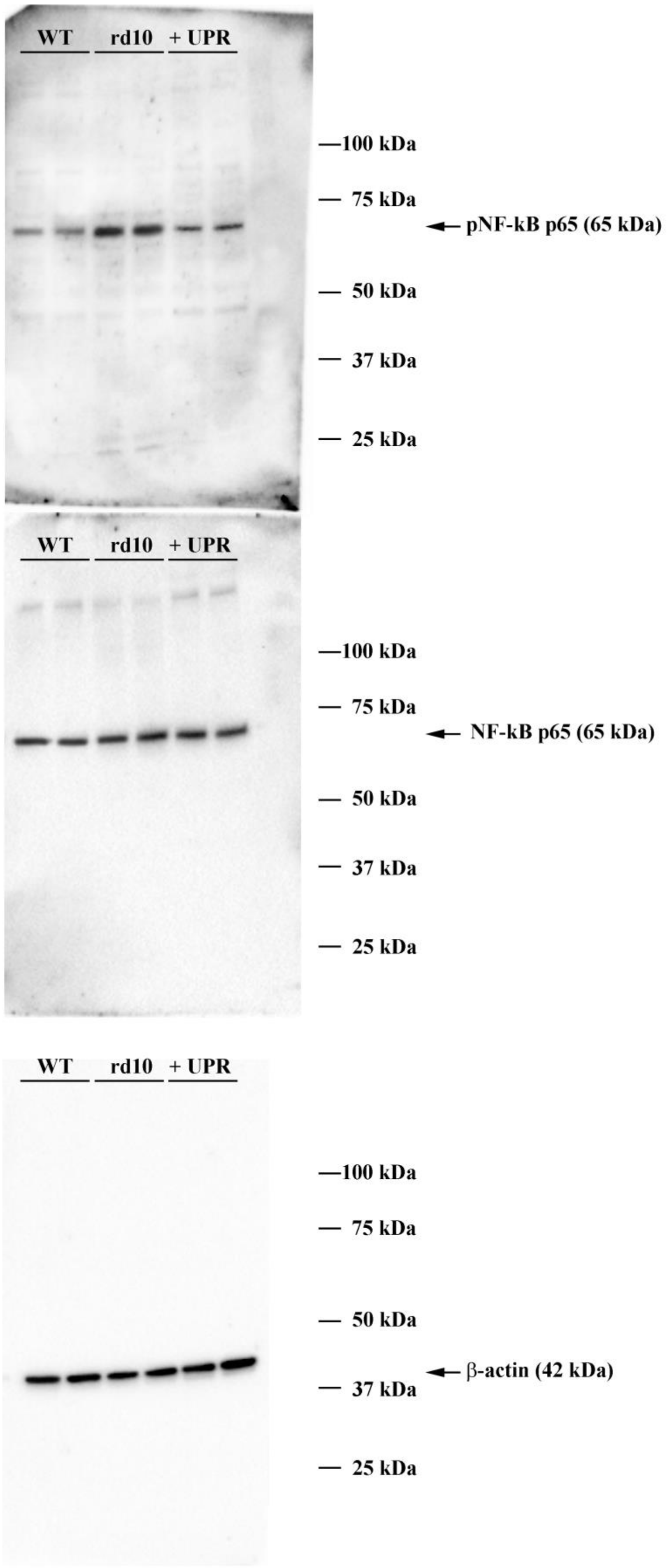

iNOS/β-actin (Fig. 1A)

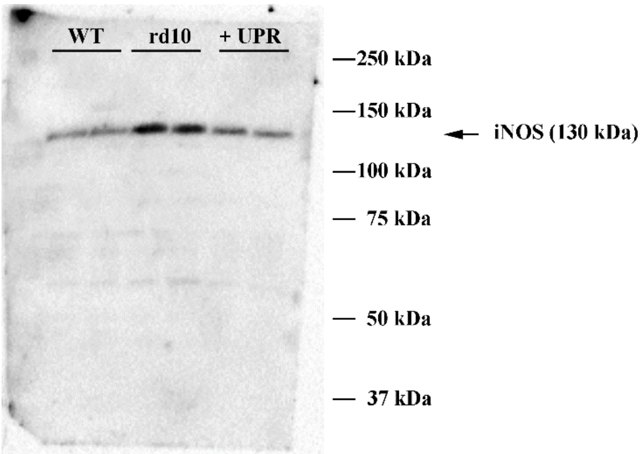

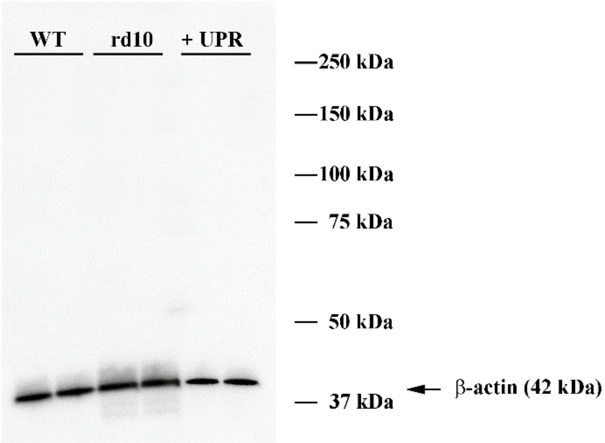

ICAM-1/β-actin (Fig. 1A)

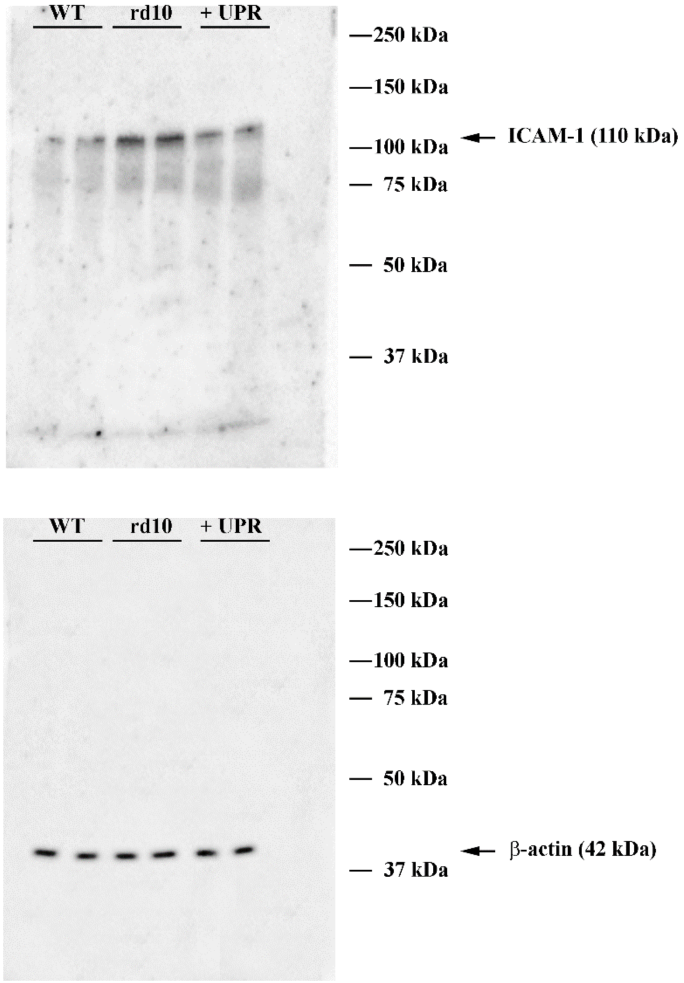

TNF-α/β-actin (Fig. 1A)

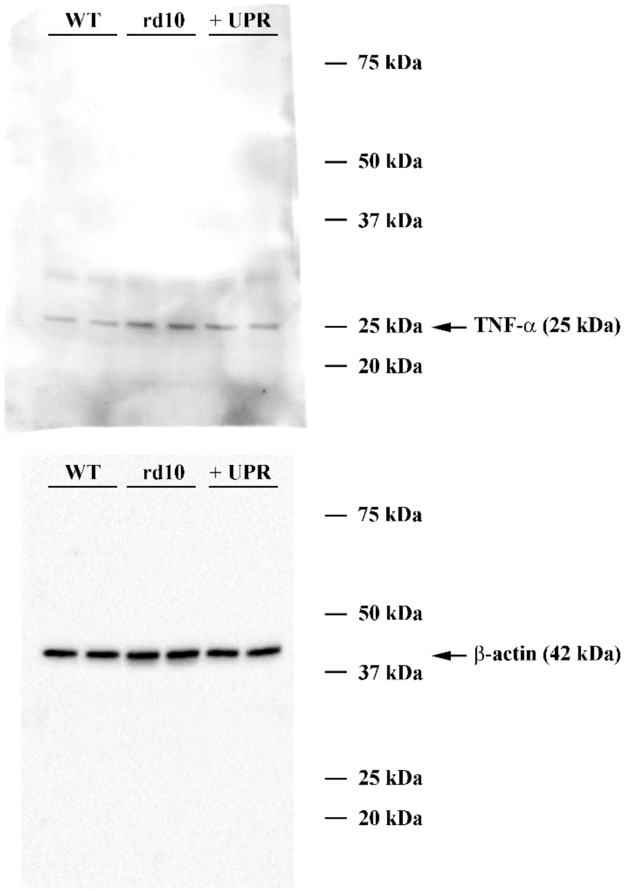

IL-6/β-actin (Fig. 1A)

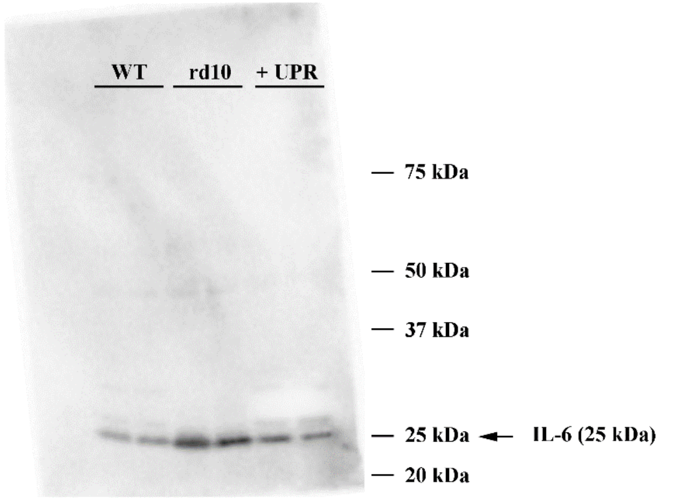

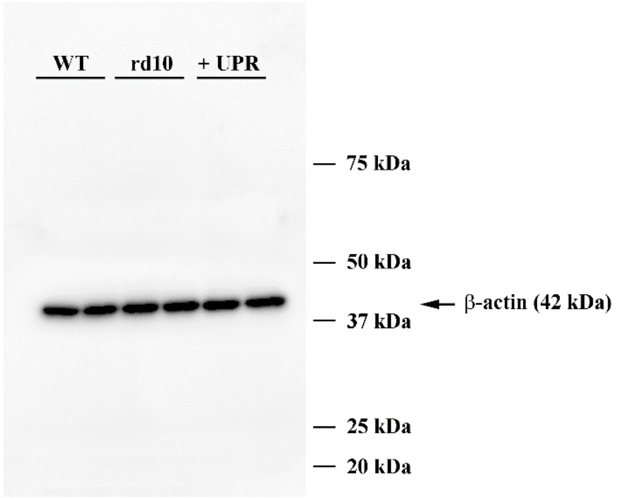

Bax/β-actin (Fig. 2A)

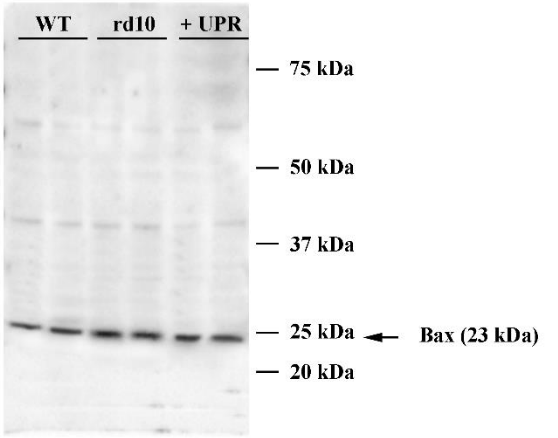

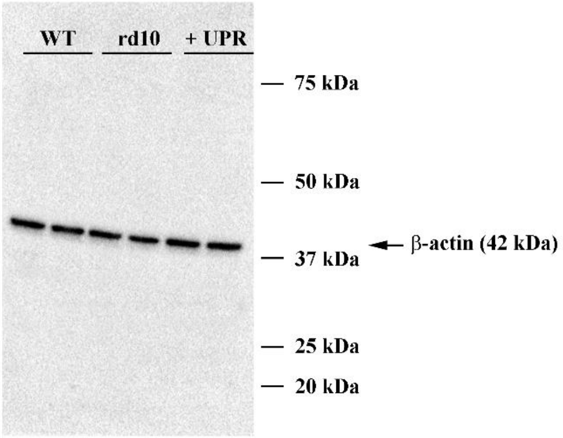

Bcl2/β-actin (Fig. 2A)

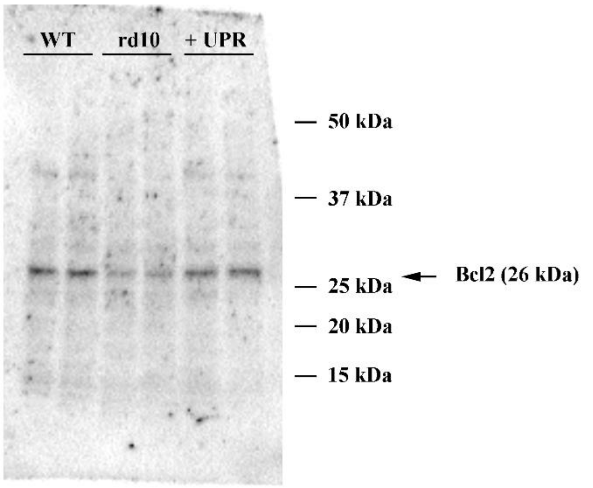

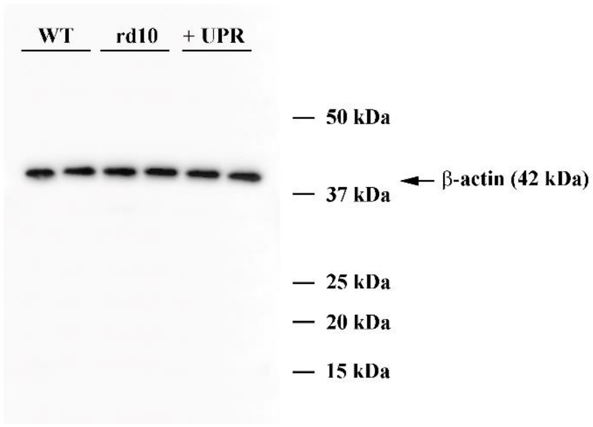

caspase 3/β-actin (Fig. 2A)

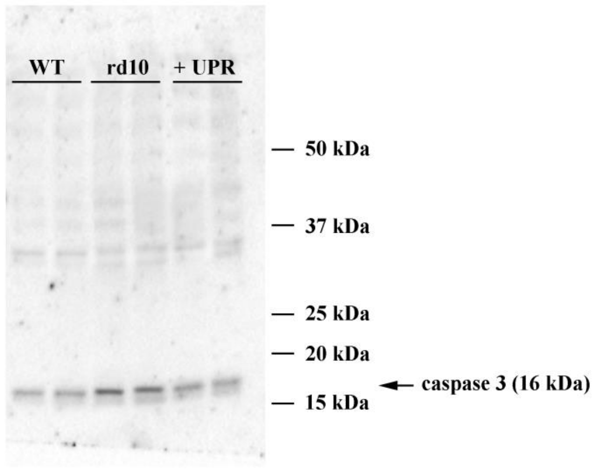

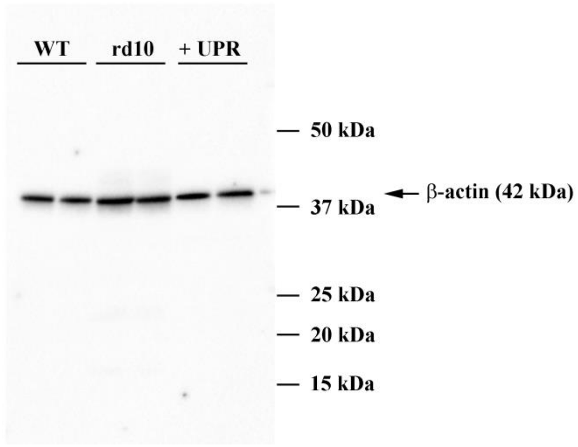

LC3 I/LC3 II/β-actin (Fig. 2D)

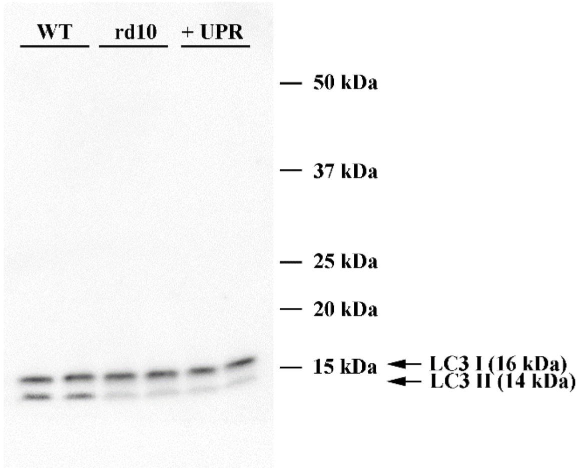

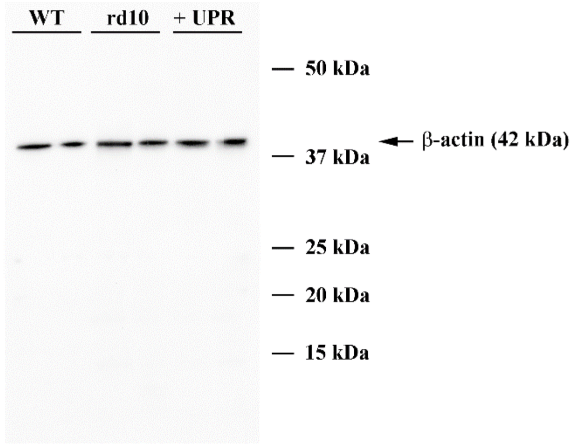

p62/β-actin (Fig. 2D)

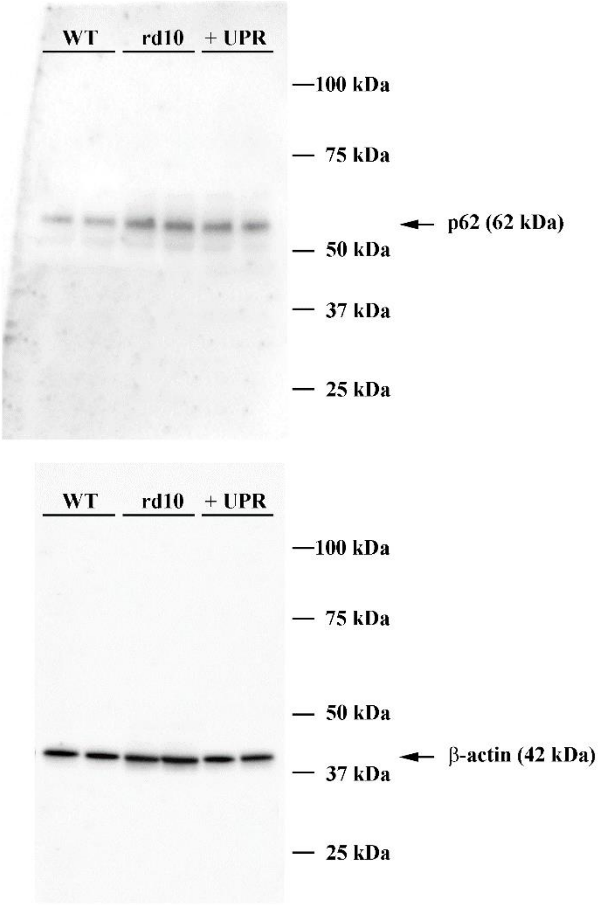

rhodopsin/β-actin (Fig. 4A)

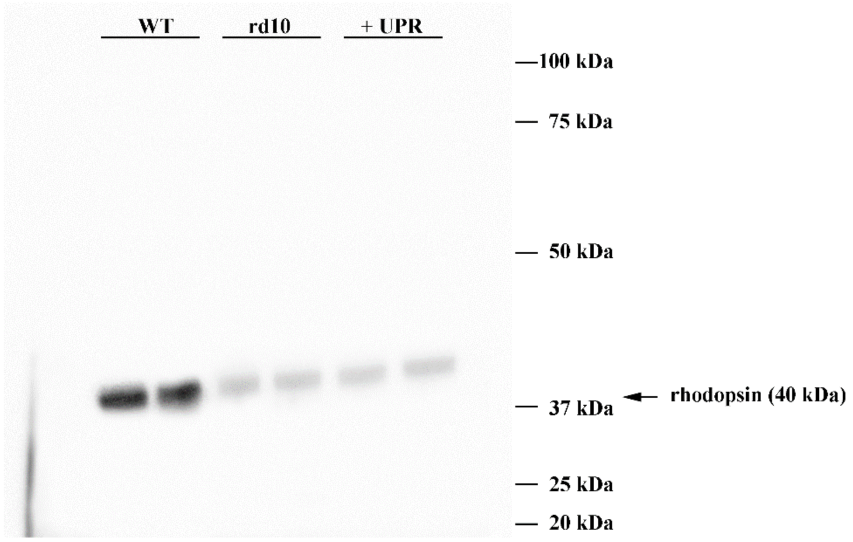

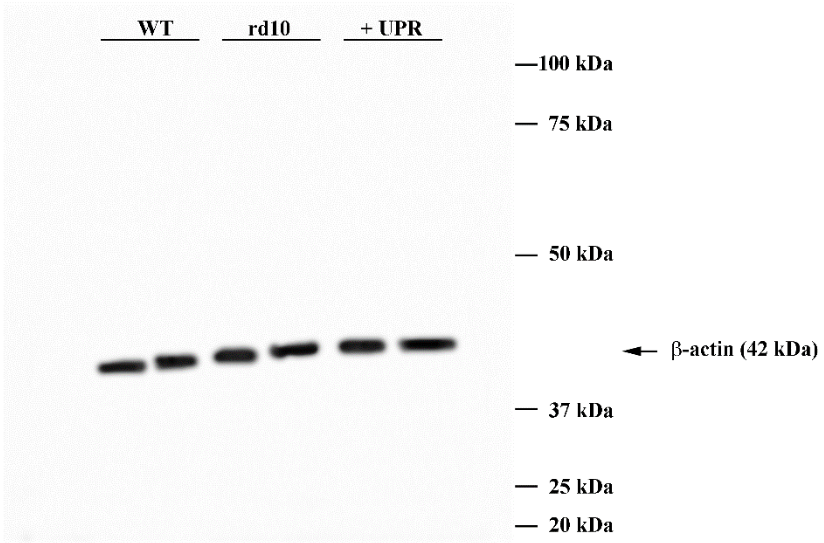

Transducin α/β-actin (Fig. 4B)

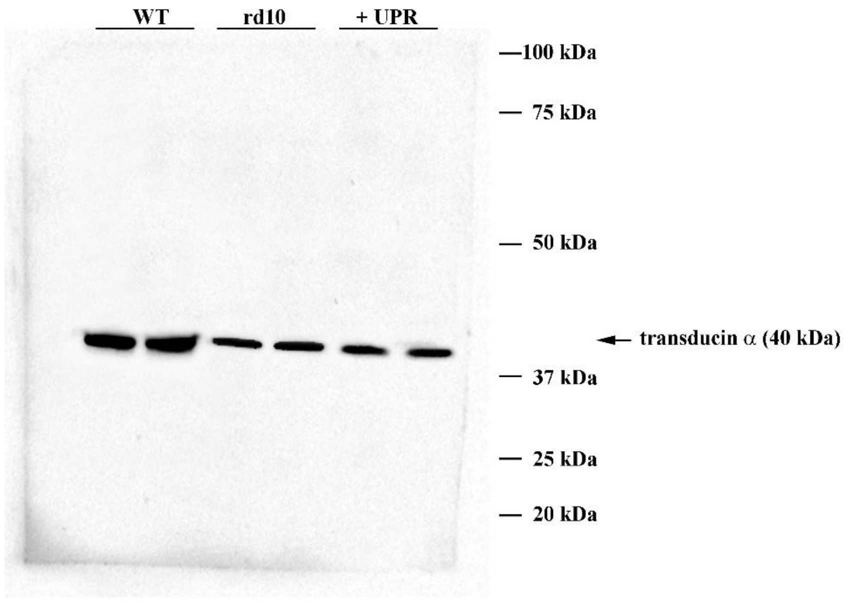

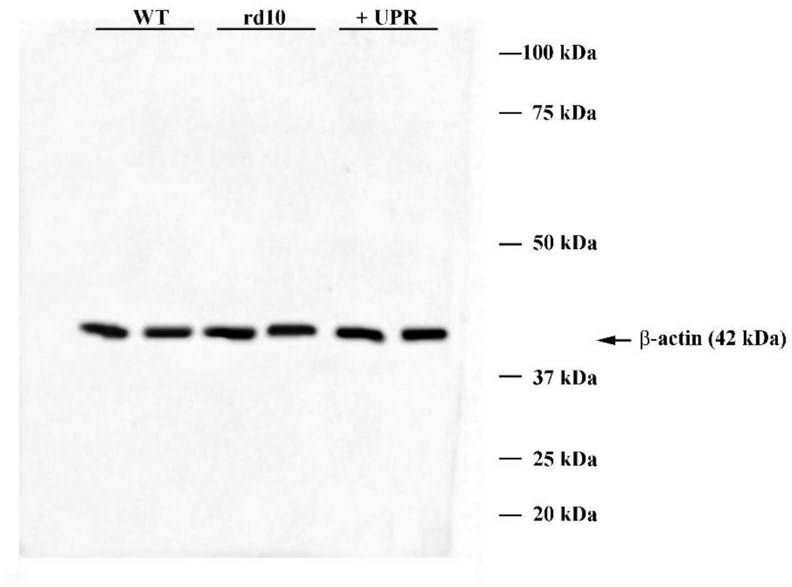

cone arrestin/β-actin (Fig. 4E)

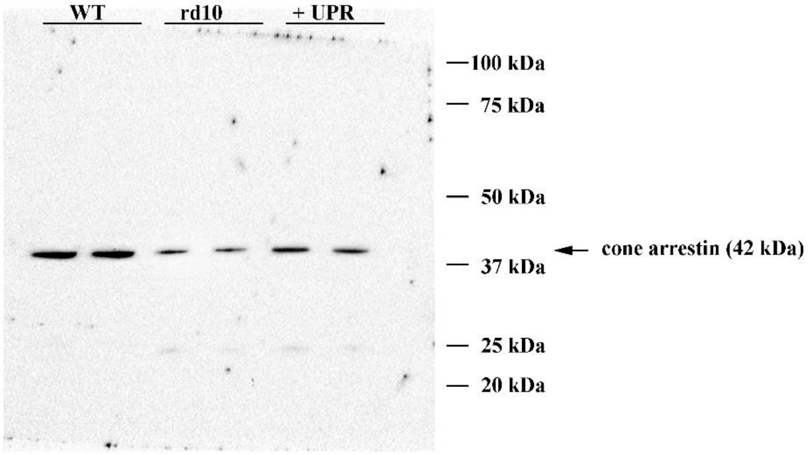

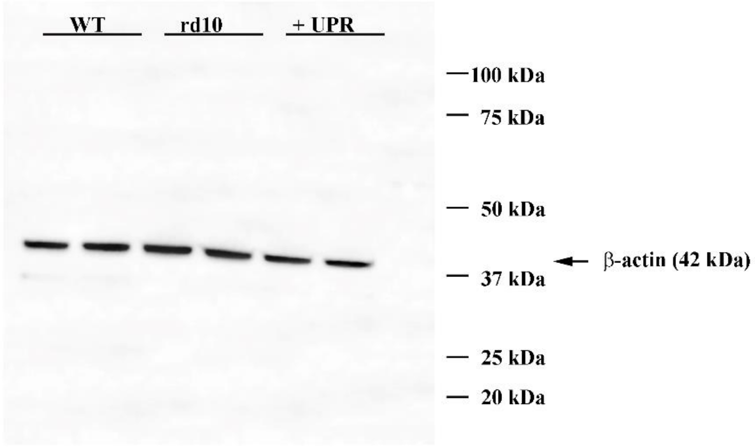

uPA/β-actin (Fig. 6)

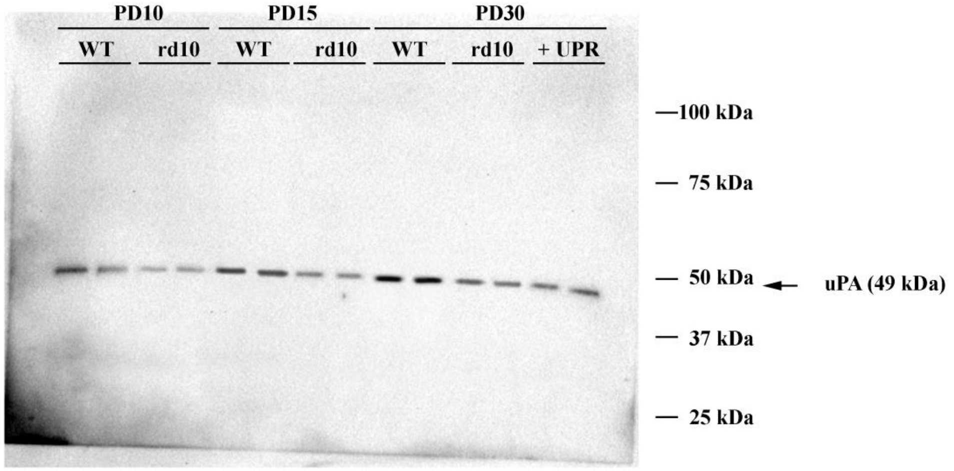

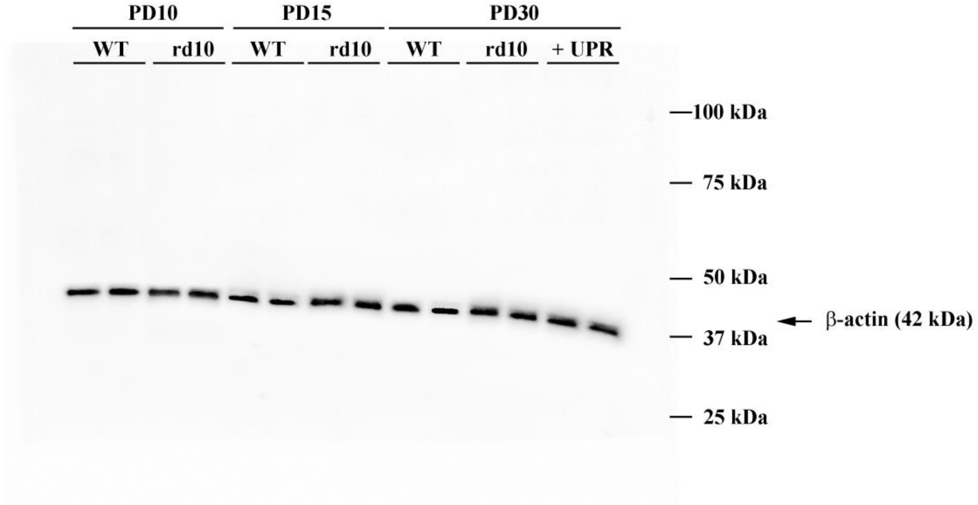

uPAR/β-actin (Fig. 6)

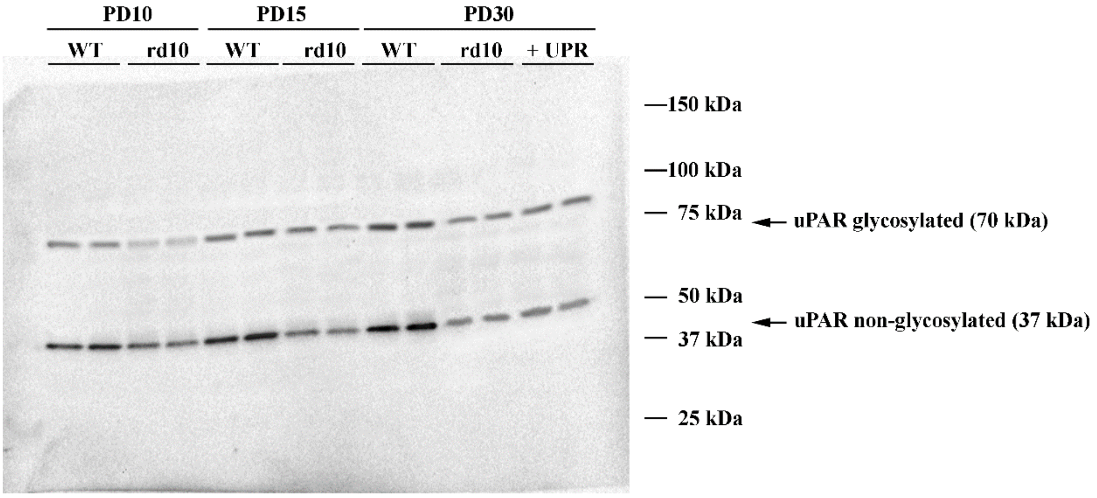

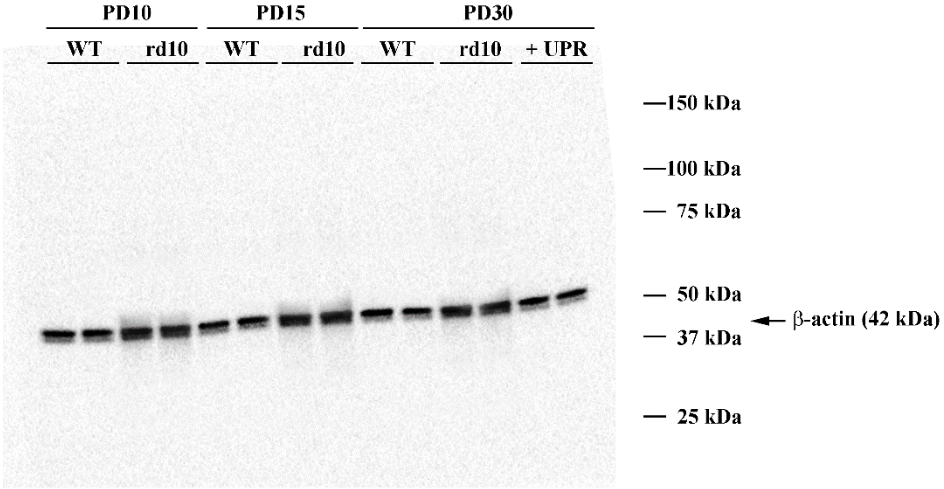

FPR1/β-actin (Fig. 6)

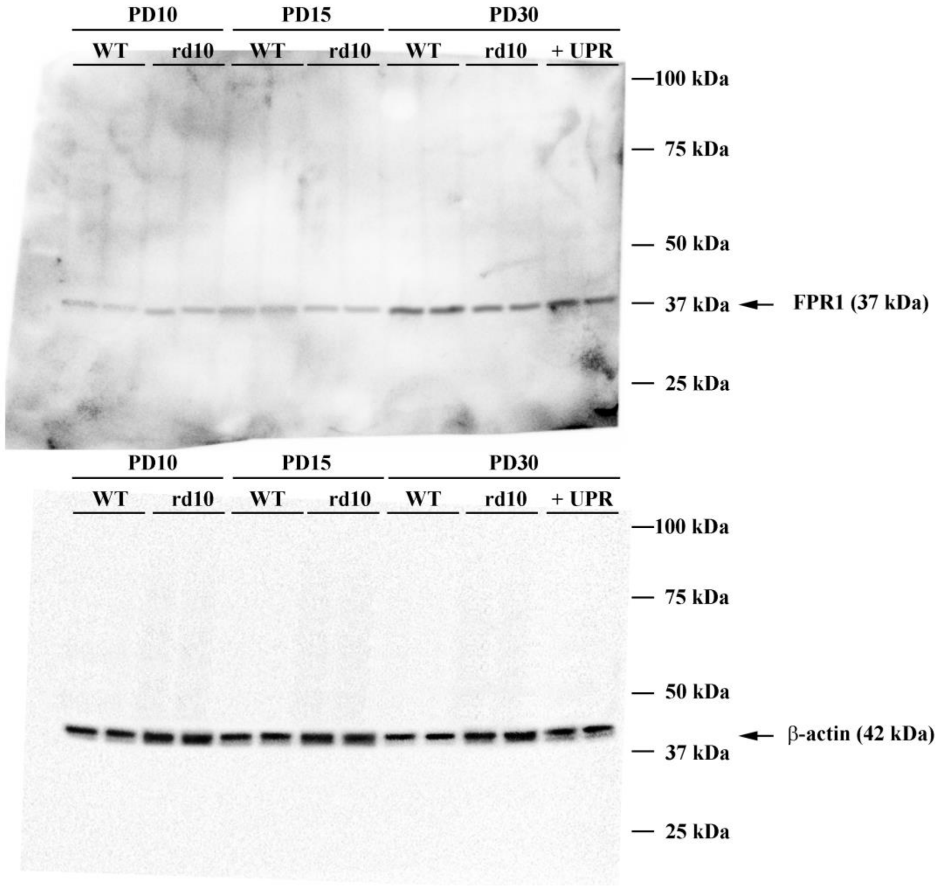

FPR2/β-actin (Fig. 6)

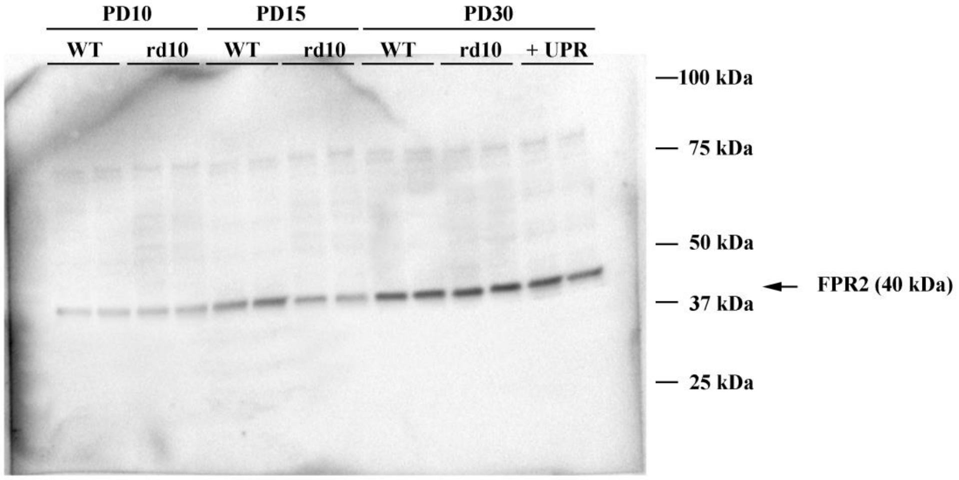

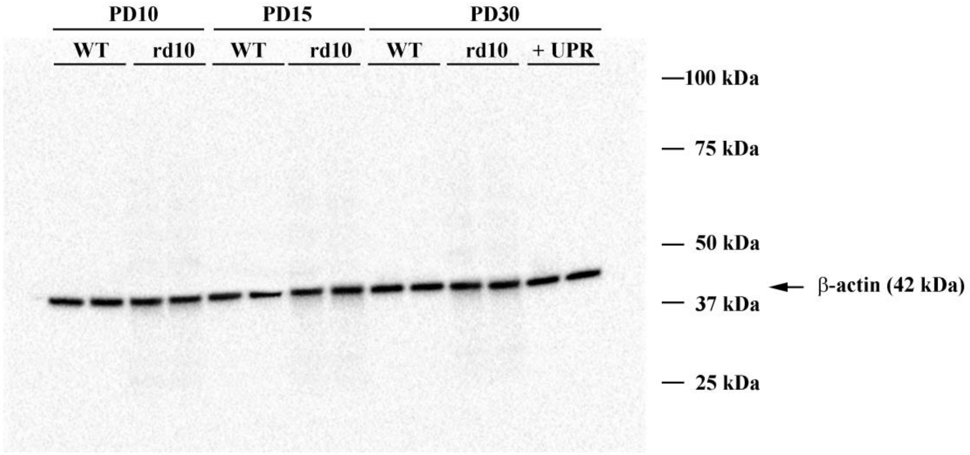

FPR3/β-actin (Fig. 6)

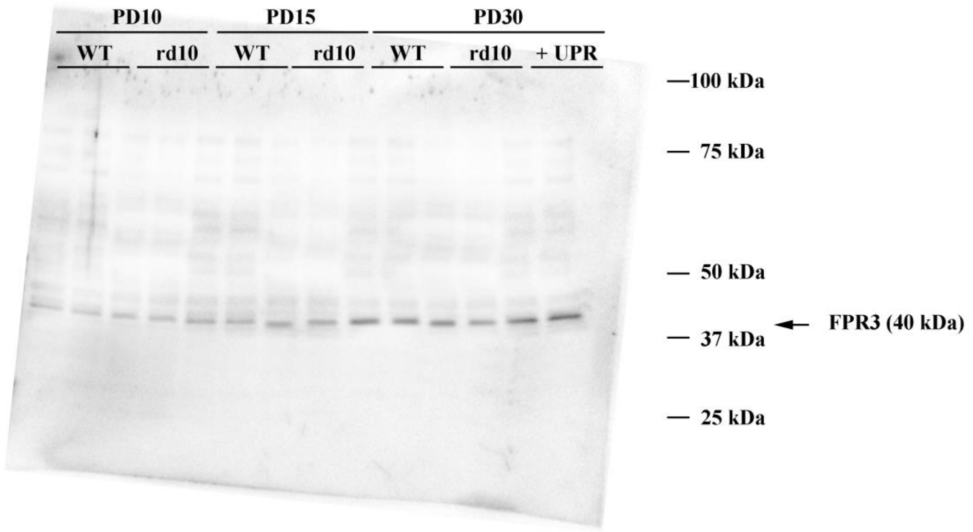

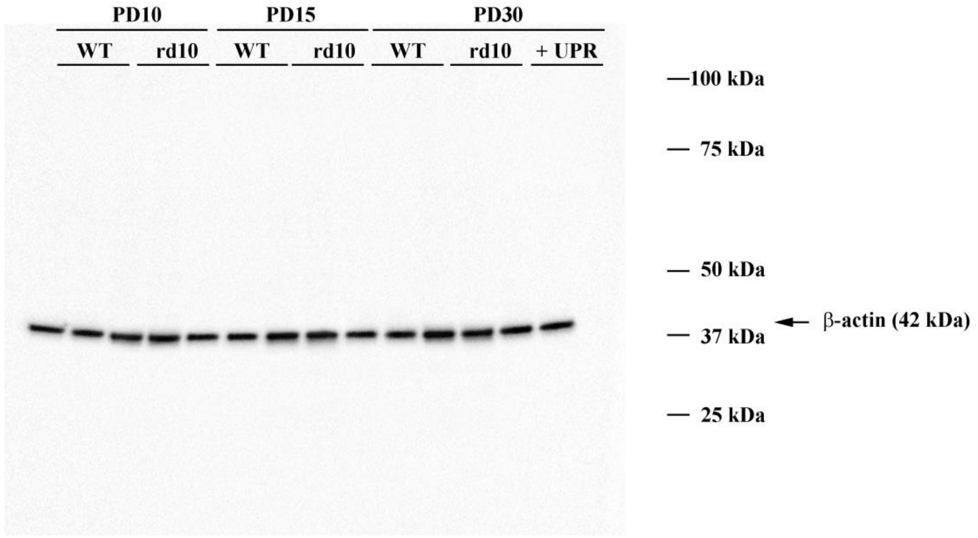

pβ3 integrin/αvβ3 integrin/β-actin (Fig. 6)

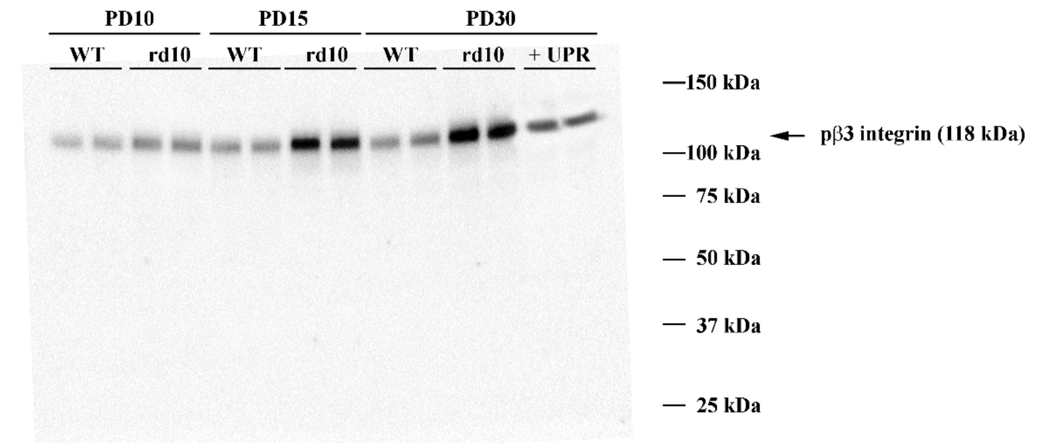

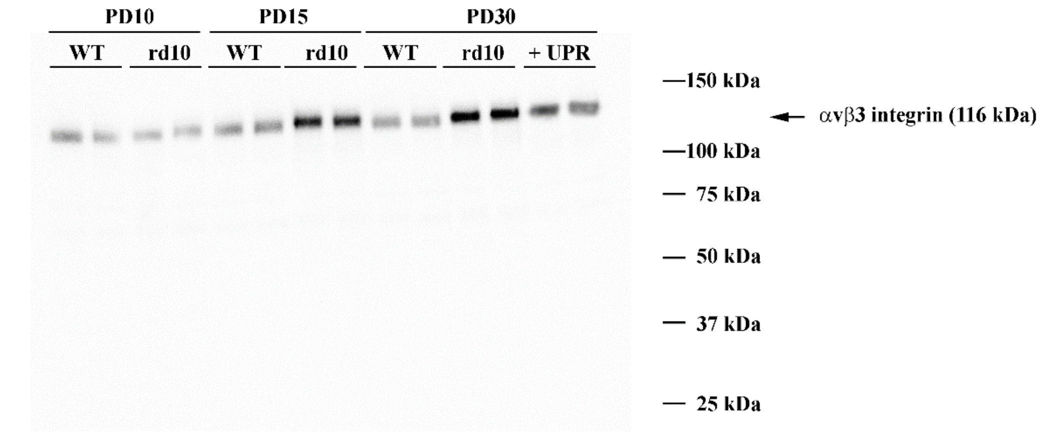

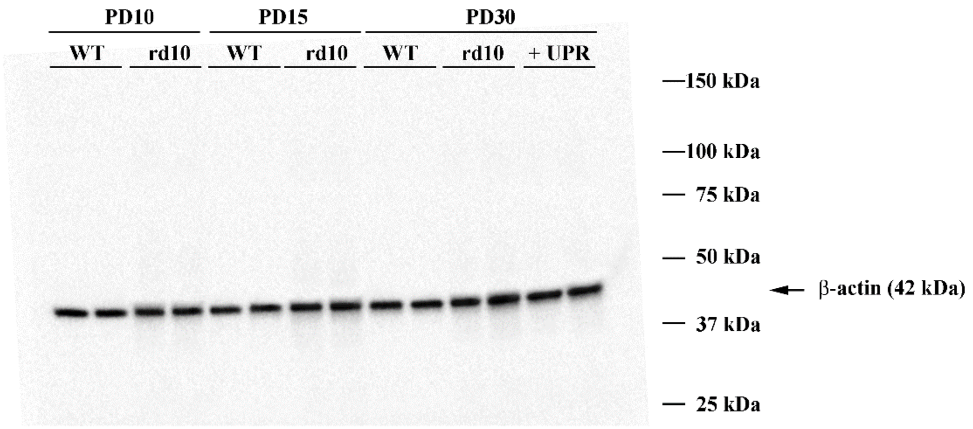

Rac1-GTP/Rac1/β-actin (Fig. 6)

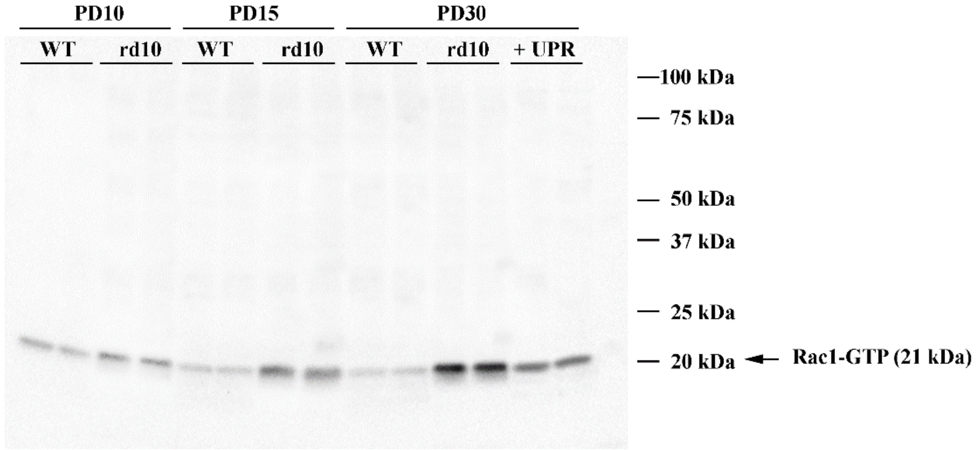

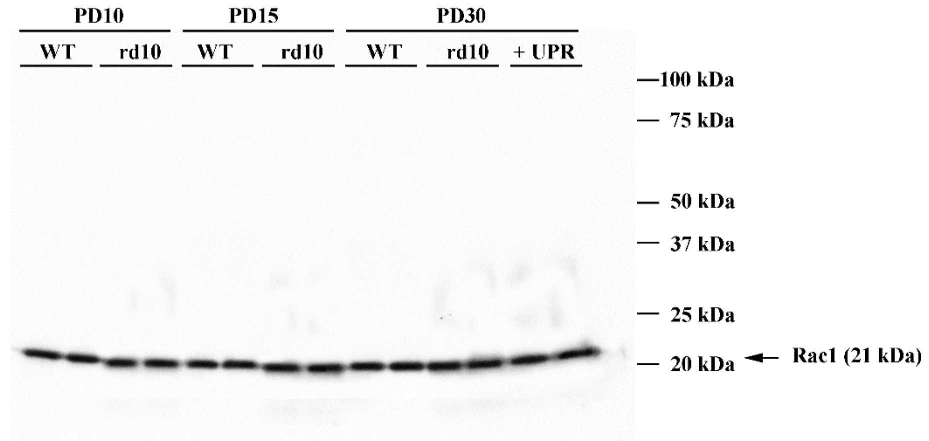

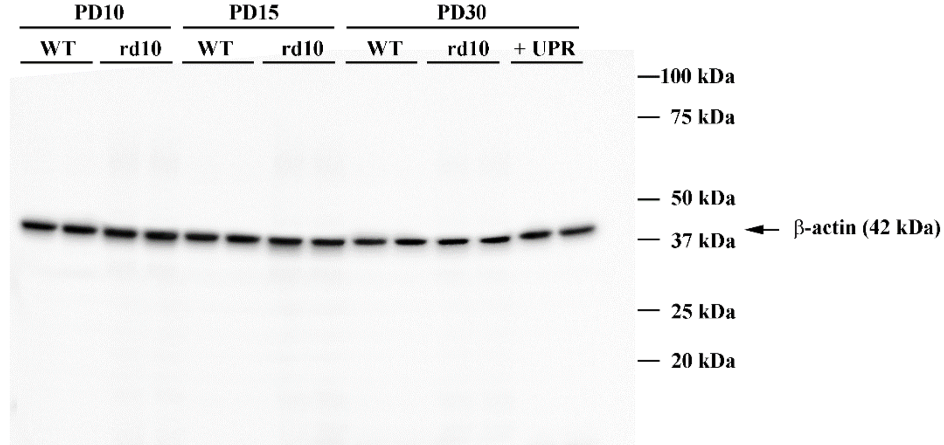

Supplement: Supplementary file 1 [file JCMM-23-5176-s001.pdf]
